# The Warburg Effect is the result of faster ATP production by glycolysis than respiration

**DOI:** 10.1073/pnas.2409509121

**Published:** 2024-11-08

**Authors:** Matthew A. Kukurugya, Saharon Rosset, Denis V. Titov

**Affiliations:** ^a^Department of Molecular & Cell Biology, University of California, Berkeley, CA 94720; ^b^Center for Computational Biology, University of California, Berkeley, CA 94720; ^c^Department of Statistics and Operations Research, Tel Aviv University, Tel Aviv 69978, Israel; ^d^Department of Nutritional Sciences & Toxicology, University of California, Berkeley, CA 94720

**Keywords:** Warburg Effect, cancer metabolism, energy metabolism, modeling, systems biology

## Abstract

Many prokaryotic and eukaryotic cells metabolize glucose to organism-specific by-products instead of fully oxidizing it to carbon dioxide and water—a phenomenon referred to as the Warburg Effect. The benefit to a cell is not fully understood, given that partial metabolism of glucose yields an order of magnitude less adenosine triphosphate (ATP) per molecule of glucose than complete oxidation. Here, we test a previously formulated hypothesis that the benefit of the Warburg Effect is to increase ATP production rate by switching from high-yielding respiration to faster glycolysis when excess glucose is available and respiration rate becomes limited by proteome occupancy. We show that glycolysis produces ATP faster per gram of pathway protein than respiration in *Escherichia coli*, *Saccharomyces cerevisiae*, and mammalian cells. We then develop a simple mathematical model of energy metabolism that uses five experimentally estimated parameters and show that this model can accurately predict absolute rates of glycolysis and respiration in all three organisms under diverse conditions, providing strong support for the validity of the ATP production rate maximization hypothesis. In addition, our measurements show that mammalian respiration produces ATP up to 10-fold slower than respiration in *E. coli* or *S. cerevisiae*, suggesting that the ATP production rate per gram of pathway protein is a highly evolvable trait that is heavily optimized in microbes. We also find that *E. coli* respiration is faster than fermentation, explaining the observation that *E. coli*, unlike *S. cerevisiae* or mammalian cells, never switch to pure fermentation in the presence of oxygen.

## Significance

Many prokaryotic and eukaryotic cells metabolize glucose to organism-specific by-products instead of fully oxidizing it to carbon dioxide and water—a phenomenon referred to as the Warburg Effect. The benefit to a cell is not fully understood, given that partial metabolism of glucose yields an order of magnitude less ATP per molecule of glucose than complete oxidation. Here, we use a combination of experiments and mathematical modeling to provide strong support for a previously formulated hypothesis that the benefit of the Warburg Effect is to increase ATP production rate by switching from high-yielding respiration to faster glycolysis when excess glucose is available and respiration rate becomes limited by proteome occupancy.

The Warburg Effect remains one of the most well-documented, yet incompletely understood phenomena in metabolism and cancer biology. In 1924, Otto Warburg made the observation that in vivo tumors preferred converting glucose to lactic acid via fermentation in the presence of oxygen instead of complete oxidation to carbon dioxide and water (1, 2). Mammalian respiration yields more than ten times more adenosine triphosphate (ATP) per molecule of glucose than fermentation. The rationale for why cells prefer lower ATP-yielding fermentation for ATP production in the presence of oxygen is not fully understood.

Although the initial discovery was made in tumor cells, Warburg Effect–like metabolism has since been observed for numerous proliferating cells, including acetate production through the phosphotransacetylase-acetate kinase (Pta-AckA) pathway in *Escherichia coli,* ethanol fermentation in *Saccharomyces cerevisiae,* and lactic acid fermentation in nontransformed mammalian cells. This phenomenon is also referred to as the Crabtree Effect in *S. cerevisiae* and “overflow metabolism” in *E. coli*. To simplify nomenclature, we will collectively refer to various organism-specific pathways for partial metabolism of glucose (i.e., fermentation in *E. coli*, *S. cerevisiae*, and mammalian cells and the respiro-fermentative Pta-AckA acetate pathway in *E. coli*) as *glycolysis*, to organism-specific pathways for complete oxidation of glucose to carbon dioxide and water as *respiration*, and to the preference for incomplete oxidation of glucose in the presence of oxygen as *the Warburg Effect*.

No unifying hypothesis is widely accepted to explain the occurrence of the Warburg Effect (3–6) in a variety of organisms. Otto Warburg himself proposed that high glycolysis rates in cancer cells were due to an impairment in respiration (2). However, numerous studies have since demonstrated a prominent role of respiration in ATP production during cell proliferation (7, 8). Other proposed mechanisms for why the Warburg Effect occurs include satisfying biosynthetic demand (9, 10), regulating redox balance (11), limiting reactive oxygen species (12, 13), allowing for faster growth in hypoxic conditions (14), increasing the ATP production rate (15, 16), outcompeting other microorganisms for resources (17–19), and optimizing metabolism under the constraints of the proteome space (20–23), mitochondrial respiratory capacity (24–26), or bacterial plasma membrane area (18, 19).

We focus on testing the previously formulated hypothesis that the Warburg Effect allows cells to maximize ATP production rate (15, 16). There is strong evolutionary pressure for cells and organisms to be able to generate ATP at a maximal rate under given conditions (15, 16). For example, unicellular organisms can grow faster than competition, cells in multicellular organisms can divide faster to speed up the immune response and wound healing, and multicellular organisms can move faster to evade predators or catch prey if they can generate ATP at a higher rate. To produce ATP at a faster rate, cells can either use a high-yield biochemical pathway that produces more ATP per molecule of glucose or a high-rate pathway that consumes more molecules of glucose per unit of time (15, 16). The switch to a high-rate pathway can only be beneficial when there is some constraint that prevents cells from simply increasing the activity of a high-yield pathway. Such a constraint can come from a finite proteome space that can be allocated to ATP-producing pathways while allowing for the expression of other pathways such as translation, biosynthesis, protein folding machinery, cytoskeleton components, etc. Here, our work builds on the resource allocation models of microbial growth (20–24). Several groups have proposed that microbes maximize their growth rate under different conditions by optimally allocating their proteome to translation, biosynthesis, energy production, and other processes. Resource allocation models are based on the observation that intracellular protein concentration is relatively stable (27, 28) and, thus, to increase the expression of one pathway, another pathway’s expression must be decreased. Within the resource allocation framework, glycolysis has been proposed to be more proteome efficient than respiration, and, therefore, switching from respiration to glycolysis allows microbial cells to either increase their allocation to ATP-producing enzymes or allocate more of their proteome to ribosomes and other biosynthetic enzymes (20, 22) to grow faster.

Here, we extend the resource allocation models to mammalian cells and provide a simple unifying explanation for the occurrence of Warburg Effect–like metabolism in *E. coli*, *S. cerevisiae*, and mammalian cells. Specifically, we test the hypothesis that the Warburg Effect allows cells to produce ATP at a maximal rate depending on glucose availability and irrespective of cell growth. At low glucose availability, respiration produces ATP faster due to its higher yield, while at high glucose availability, glycolysis produces ATP faster due to its higher rate. To test this hypothesis, we developed a coarse-grained mathematical model that predicts the occurrence of the Warburg Effect at specific values of five biochemical parameters, including yield and specific activity of respiration and glycolysis, and maximal proteome fraction that can be occupied by ATP-producing enzymes. Our model has a unique analytical solution and no adjustable parameters, allowing for the direct prediction of experimental results. We experimentally estimated the values of the five biochemical parameters for each organism. Using these biochemical parameters, we show that our model quantitatively predicts the onset of the Warburg Effect, as well as glycolytic and respiration rates under various conditions in *E. coli*, *S. cerevisiae*, and mammalian cells, irrespective of growth rate. The ability of our model to predict the results of experiments in diverse organisms and conditions and without adjustable parameters provides strong evidence for the validity of this hypothesis.

## Results

### The Warburg Effect Emerges from a Simple Model of Energy Metabolism That Maximizes ATP Production Rate.

**Fig. 1.**
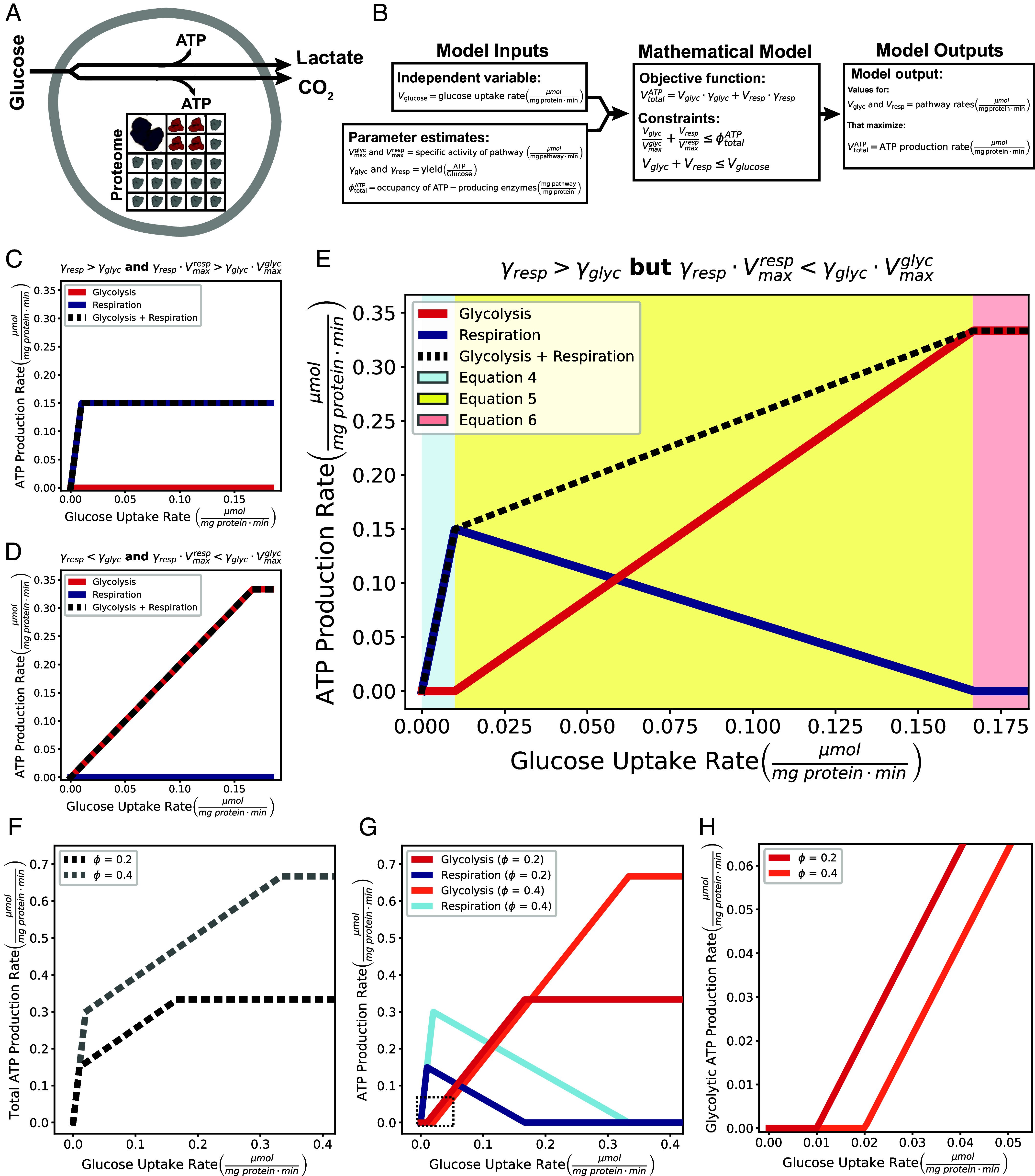
The Warburg Effect emerges from a simple model of energy metabolism that maximizes ATP production rate. (*A*) Illustration of the model. (*B*) Overview of the mathematical model. (*C*) Preferred ATP-producing pathways if parameters of yield of ATP per molecule of glucose (γ) and the specific activity of ATP production (γ∙V) are both higher for respiration, (*D*) both higher for glycolysis, or (*E*) if the yield is higher for respiration ($\gamma_{\mathrm{resp}}>\gamma_{\mathrm{glyc}}$), but the specific activity of ATP production is higher for glycolysis ($\gamma_{\mathrm{resp}}∙V_{\max}^{\mathrm{resp}}<\gamma_{\mathrm{glyc}}∙V_{\max}^{\mathrm{glyc}}$). In each case, the total ATP production rate is represented by the dashed line. (*F*) Increasing the proteome space dedicated to ATP-producing enzymes increases ATP production rate across glucose uptake rates and (*G*) delays the switch from respiration to glycolysis. The box in Fig. 1*G* is shown in (*H*) to highlight the delayed onset of glycolysis.

We test a hypothesis that the Warburg Effect results from cells switching between glycolysis and respiration to achieve maximal ATP production rates at different environmental conditions. The ATP production rate is controlled to meet cellular ATP demand. In proliferating cells, ATP demand correlates with growth as it supports biosynthesis. In differentiated cells, ATP demand can increase from nongrowth activities, such as muscle contraction or biosynthesis of macromolecules for secretion. We assume cells obey three biochemical constraints while optimizing ATP production (Fig. 1*A*): i) Glucose consumption is limited by either its availability in the surrounding media or by glucose uptake capacity of the cell. We assume that oxygen is present at saturating levels, but this assumption is not required, as we describe at the end of this section. ii) A limited fraction of the proteome can be allocated to ATP-producing enzymes. We assume that cells are free to shift the relative rates of glycolysis and respiration through a combination of changes in the expression of enzymes and transporters, posttranslational modifications, and allosteric regulation as long as the sum of glycolysis and respiration enzymes stays within the proteome fraction allocated to ATP-producing enzymes. iii) The maximal ATP production rate by either glycolysis or respiration pathways is limited by the maximal activity of respective enzymes. To describe the maximal ATP production rate by glycolysis or respiration, we utilize a metric that we call the specific activity of the pathway. The specific activity of the pathway (*V_max_*) is defined as the µmol of substrate per minute per milligram of pathway protein. This metric is analogous to the widely used specific activity of enzymes and has also been referred to as proteome efficiency in previous studies (14, 22). We will use specific activities of glucose consumption and ATP production, where the latter is obtained by multiplying the former by the ATP yield per glucose of the relevant pathway.

To test the feasibility of our hypothesis, we constructed a mathematical model (Fig. 1*B*). Our aim was to keep the mathematical model as simple as possible to keep the interpretation of results straightforward. Our model calculates the cellular rates of glycolysis (*V_glyc_*) and respiration (*V_resp_*) that yield maximal ATP production rate (*V_ATP_*) at a given glucose uptake rate (*V_glucose_*), which are all in units of µmol per minute per milligram of cellular protein. The model uses five biochemical parameters, including the ATP yield ($\gamma_{\mathrm{glyc}}$ and $\gamma_{\mathrm{resp}}$) and specific activity of glycolysis and respiration ($V_{\max}^{\mathrm{glyc}}$ and $V_{\max}^{\mathrm{resp}}$) and fraction of the proteome that is occupied by ATP-producing enzymes ($\phi_{\mathrm{total}}^{\mathrm{ATP}}$). The model must also satisfy two simple constraints that i) the rate of glycolysis and respiration cannot proceed faster than what is allowed by the proteome allocation for ATP-producing enzymes $\left(\phi_{\mathrm{total}}^{\mathrm{ATP}}\right)$ and ii) glucose uptake rate cannot be larger than the combined rates of glycolysis and respiration.

The model can be written as an optimization problem:

[1] $$Maximize:\;V_{\mathrm{ATP}}=V_{\mathrm{glyc}}∙\gamma_{\mathrm{glyc}}+V_{\mathrm{resp}}∙\gamma_{\mathrm{resp}},$$

[2] $$Subject\;to:\;\frac{V_{\mathrm{glyc}}}{V_{\max}^{\mathrm{glyc}}}+\frac{V_{\mathrm{resp}}}{V_{\max}^{\mathrm{resp}}}\leq \phi_{\mathrm{total}}^{\mathrm{ATP}},$$

[3] $$V_{\mathrm{glyc}}+V_{\mathrm{resp}}\leq V_{\mathrm{glucose}}.$$

The model can either be solved numerically using linear programming, which guarantees identification of a global maximum, or analytically using Lagrange multipliers with Karush–Kuhn–Tucker conditions (29) (see *SI Appendix* for details). The solution predicts that if one pathway has both a greater yield of ATP per molecule of glucose (γ) and a higher specific activity of ATP production (V∙γ), that pathway allows for maximal ATP production rate, independent of the glucose uptake rate (Fig. 1 *C* and *D*). However, if the yield is greater for one pathway (e.g., respiration such that $\gamma_{\mathrm{glyc}}<\gamma_{\mathrm{resp}}$), but the specific activity of ATP production is higher for the other pathway (e.g., glycolysis such that $V_{\max}^{\mathrm{glyc}}∙\gamma_{\mathrm{glyc}}>V_{\max}^{\mathrm{resp}}∙\gamma_{\mathrm{resp}}$), each pathway allows for maximal ATP production rate depending on glucose availability (Fig. 1*E*). The analytical solution of the model with respect to the glucose availability assuming that $\gamma_{\mathrm{glyc}}<\gamma_{\mathrm{resp}}$ and $V_{\max}^{\mathrm{glyc}}∙\gamma_{\mathrm{glyc}}>V_{\max}^{\mathrm{resp}}∙\gamma_{\mathrm{resp}}$ is given by the following equations:

[4] $$\mathrm{If}\;V_{\max}^{\mathrm{resp}}\cdot \phi_{\mathrm{total}}^{\mathrm{ATP}}>V_{\mathrm{glucose}}\;\text{then}\;V_{\mathrm{glyc}}=0,V_{\mathrm{resp}}=V_{\mathrm{glucose}}.$$

[5] $$\begin{array}{c} \mathrm{If}\;\;\phi_{\mathrm{total}}^{\mathrm{ATP}}∙V_{\max}^{\mathrm{glyc}}>>V_{\mathrm{glucose}}>>\phi_{\mathrm{total}}^{\mathrm{ATP}}∙V_{\max}^{\mathrm{resp}}\\ \mathrm{then}\;V_{\mathrm{glyc}}=\frac{\phi_{\mathrm{total}}^{\mathrm{ATP}}∙V_{\max}^{\mathrm{glyc}}∙V_{\max}^{\mathrm{resp}}-V_{\mathrm{glucose}}∙V_{\max}^{\mathrm{glyc}}}{V_{\max}^{\mathrm{resp}}-V_{\max}^{\mathrm{glyc}}},\\ \;V_{\mathrm{resp}}=\frac{V_{\max}^{\mathrm{resp}}(\phi_{\mathrm{total}}^{\mathrm{ATP}}∙V_{\max}^{\mathrm{glyc}}∙V_{\max}^{\mathrm{resp}}-V_{\mathrm{glucose}})}{V_{\max}^{\mathrm{glyc}}-V_{\max}^{\mathrm{resp}}}. \end{array}$$

[6] $$\mathrm{If}\;V_{\max}^{\mathrm{resp}}∙\phi_{\mathrm{total}}^{\mathrm{ATP}}<V_{\mathrm{glucose}}\;\mathrm{then}\;V_{\mathrm{glyc}}=V_{\max}^{\mathrm{glyc}}\cdot \phi_{\mathrm{total}}^{\mathrm{ATP}},V_{\mathrm{resp}}=0.$$

Eqs. **4**–**6** predict that only respiration is used at low glucose uptake rates when $V_{\max}^{\mathrm{resp}}\cdot \phi_{\mathrm{total}}^{\mathrm{ATP}}>V_{\mathrm{glucose}}$ (Eq. **4**); respiration proves beneficial when glucose is limited as it maximizes ATP production per glucose molecule (Fig. 1*E*, blue shaded region). As the glucose uptake rate increases, the limiting factor shifts from substrate availability to the capacity of the proteome ($\phi_{\mathrm{total}}^{\mathrm{ATP}}$) (Fig. 1*E*). When the proteome allocation to ATP-producing enzymes is filled by respiratory enzymes, glycolysis can be substituted for respiration to further increase the ATP production rate since it generates ATP more rapidly per unit of protein (Fig. 1*E*, yellow shaded region). The rate of this trade-off is defined by Eq. **5**. The shift from respiration to glycolysis occurs as a gradual substitution until only glycolysis is utilized; glycolysis is favored at high glucose uptake rates when $V_{max}^{\;glyc}∙\phi_{total}^{ATP}<V_{glucose}$ (Eq. **6**) (Fig. 1*E*, red shaded region). In other words, a cell can produce ATP at the fastest rate using high-yielding respiration at low glucose availability (Fig. 1*E* and *SI Appendix*, Fig. S1 *A* and *E*), a mixture of high-yielding respiration and high rate glycolysis at intermediate glucose availability (Fig. 1*E* and *SI Appendix*, Fig. S1 *B*, *C*, *F*, and *G*), and with glycolysis alone at high glucose availability to achieve the maximal ATP production rate (Fig. 1*E* and *SI Appendix*, Fig. S1 *D* and *H*).

While the specific values of the yield and specific activity predict the benefit of switching from respiration to glycolysis, the glucose uptake rate when the switch occurs is determined by the total proteome space dedicated to ATP-producing enzymes ($\phi_{\mathrm{total}}^{\mathrm{ATP}}$) (Eq. **2**). Without the proteome constraint, cells would always be able to express more respiratory proteins to increase ATP production without compromising on ATP yield. Increasing the proteome fraction dedicated to ATP-producing enzymes ($\phi_{\mathrm{total}}^{\mathrm{ATP}}$) increases the capacity for ATP production across glucose uptake rates (Fig. 1*F*) and delays the switch from respiration to glycolysis (Fig. 1 *G* and *H*).

Our simple model assumes that glucose is used as the substrate for both glycolysis and respiration; however, the conclusions stay the same if a respiratory substrate other than glucose is used for respiration, such as acetate, glycerol, fatty acids, or amino acids (*SI Appendix*, Fig. S2 and see *SI Appendix* for details). The latter can be intuitively understood by considering that the majority of respiratory ATP is produced by the tricarboxylic acid (TCA) cycle and electron transport chain (ETC) enzymes for physiologically relevant respiratory substrates, and thus, the specific activity of respiratory ATP production is similar for different substrates.

All data used for parameter estimation and prediction validation in the following sections were collected under oxygen-rich conditions, therefore we assume oxygen saturation in our model. However, our model can also be extended to include an oxygen consumption constraint to simulate anaerobic or hypoxic environments (see *SI Appendix* for model formulation). In these oxygen-limiting conditions, our model predicts that glycolysis will be utilized for ATP production at a lower glucose uptake rate as compared to oxygen-rich environments (*SI Appendix*, Fig S3). Furthermore, our model predicts the Pasteur Effect, where glycolysis is inhibited as oxygen becomes more available (*SI Appendix*, Fig S3) (30) and is well known to be driven by the maximization of the ATP production rate (31–34).

In summary, our model predicts a transition from respiration to glycolysis as glucose availability increases (i.e., the Warburg Effect) under certain combinations of yield and specific activity of glycolysis and respiration.

### Glycolysis Produces ATP at a Faster Rate than Respiration.

We next estimated the yields ($\gamma_{\mathrm{glyc}}$ and $\gamma_{\mathrm{resp}}$) and specific activities of ATP production of glycolysis and respiration ($V_{\max}^{\mathrm{glyc}}∙\gamma_{\mathrm{glyc}}$ and $V_{\max}^{\mathrm{resp}}∙\gamma_{\mathrm{resp}}$) to determine whether their values fall into the range where our model predicts the occurrence of the Warburg Effect: $\gamma_{\mathrm{glyc}}<\gamma_{\mathrm{resp}}$ and $V_{\max}^{\mathrm{glyc}}∙\gamma_{\mathrm{glyc}}>V_{\max}^{\mathrm{resp}}∙\gamma_{\mathrm{resp}}$. To test the general applicability of our hypothesis, we chose to focus on three organisms, *E. coli*, *S. cerevisiae*, and mammalian cells since they span multiple kingdoms of life, exhibit a Warburg Effect–like metabolic switch, have unique bioenergetic pathways (*SI Appendix*, Fig. S4), and have been extensively studied.

We first compiled the ATP yields of each pathway per molecule of glucose (*SI Appendix*, Fig. S5 *A*–*C* and Datasets S1–S3). We found that respiration yields 10-, 8-, and 12-fold more ATP per glucose than fermentative glycolysis in *E. coli*, *S. cerevisiae*, and mammalian cells, respectively (*SI Appendix*, Fig. S5 *A*–*C*). In *E. coli*, we considered the respiro-fermentative Pta-AckA pathway as it is one of the main ATP-producing pathways in *E. coli* in the presence of both oxygen and glucose (35–37). This pathway, involving Pta and AckA, is a distinct form of glycolysis because it is not strictly fermentative. Unlike fermentative pathways, the Pta-AckA pathway uses ETC to oxidize nicotinamide adenine dinucleotide (NADH) produced by glycolysis, but unlike respiration, it does not use the TCA cycle to produce more NADH. Consequently, it requires both glycolysis and the ETC to function. This integration leads to obligate oxygen consumption and results in a higher ATP yield per glucose molecule as compared to fermentation. However, respiration in *E. coli* still yields twofold more ATP per glucose than the Pta-AckA pathway.

**Fig. 2.**
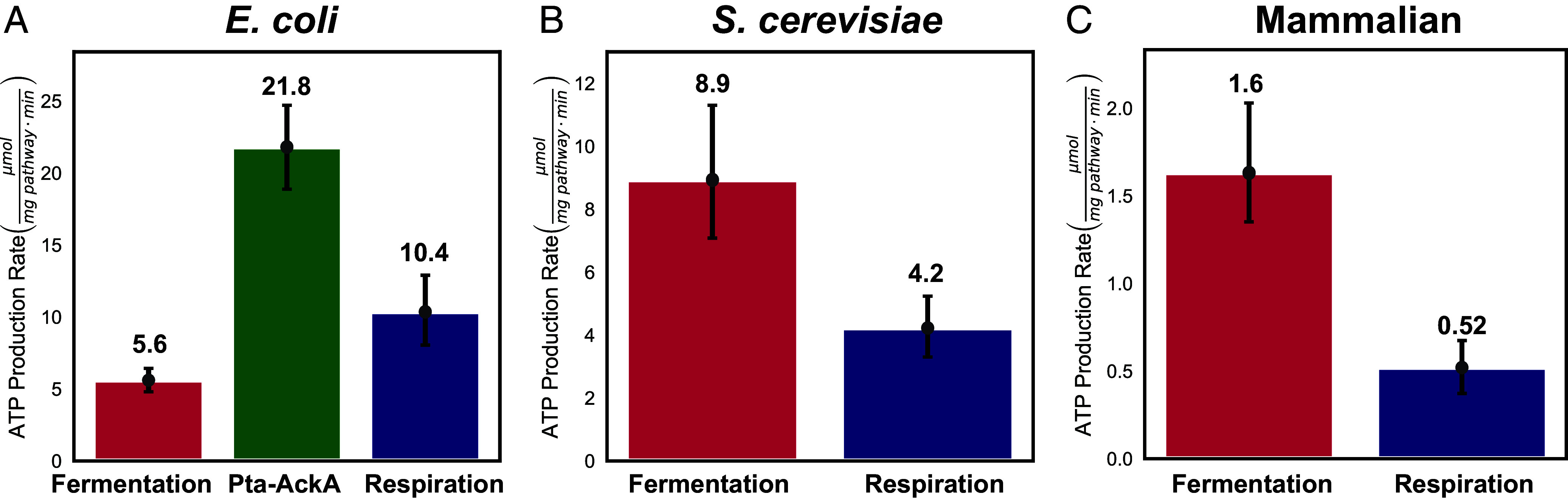
Specific activities of ATP production of relevant pathways for *E. coli*, *S. cerevisiae*, and mammalian cells. (*A*) Specific activity of ATP production (μmol mg pathway^−1^ min^−1^) for fermentation (red), the Pta-AckA pathway (green), and respiration (blue) for *E. coli*. (*B* and *C*) Specific activity of ATP production (μmol mg pathway^−1^ min^−1^) for fermentation (red) and respiration (blue) for *S. cerevisiae* and mammalian cells, respectively. Specific activity of ATP production rate is given by $V_{\max}∙\gamma$. Error bars are the 95 percent CI calculated from 10,000 bootstrap iterations.

We used experimental data to estimate the specific activities of ATP production for each pathway (V∙γ). To that end, we compiled an extensive dataset of physiological measurements to estimate the maximal rate of glycolysis and respiration per mg of cellular protein and divided those values by the fraction of the proteome occupied by glycolysis and respiration, respectively, determined from proteomics data (*SI Appendix*, Fig. S5 *D*–*I* and Datasets S1–S3). Our estimates showed that glycolysis has 0.54- (2.1- for the Pta-AckA pathway), 2.1-, and 3.1-fold faster rates per mg of pathway protein than respiration for *E. coli*, *S. cerevisiae*, and mammalian cells, respectively (Fig. 2). Our estimates allowed us to calculate the maximal ATP production rates of fermentative glycolysis and respiration in 11 transformed mammalian cell lines (*SI Appendix*, Fig. S6).

Previous studies have used different methodology to estimate the ratios of specific activities of ATP production (referred to as proteome efficiencies) of the Pta-AckA pathway and respiration in *E. coli* to be 1.92 (22) and 2.31 (23) and for fermentation and respiration in *S. cerevisiae* to be 1.66 (23) and 1.63 (38), which are all within the 95% CI of our estimates (Fig. 2 *A* and *B*). Absolute values for *E. coli* were reported (22) to be 65 and 34 μmol mg pathway^−1^ min^−1^ for Pta-AckA and respiration, respectively (converted from 750 and 390 mM per OD_600_ per hour using OD_600_ = 192.5 mg protein/L), which are the same ratio but approximately threefold higher absolute values compared to our estimates. The values of acetate secretion and percent proteome allocation of the Pta-AckA pathway appear to be similar between the latter study and our estimates. However, the difference in absolute values remains unclear and might be attributed to minor discrepancies in unit conversion. Absolute values for *S. cerevisiae* were previously estimated (38) using a genome-scale flux balance model and molecular weights of enzymes to be 4.6 and 7.5 μmol mg pathway^−1^ min^−1^ for respiration and glycolysis, respectively, which are within the 95% CI of our estimates. Finally, one study (14) found that respiration is more proteome efficient than glycolysis in *S. cerevisiae* and CD8^+^ T cells. The study reported experimental values that differ from those observed in several previous studies, including a 10-fold higher oxygen consumption in activated T cells and ~fourfold higher ratio of glycolysis to respiration proteome occupancy in *S. cerevisiae*, which likely contributed to a different conclusion (*SI Appendix*, Fig. S7).

In addition to direct experimental measurements, we estimated the molecular weight of glycolysis and respiration by summing up the molecular weights of the individual components multiplied by their stoichiometry in the relevant pathway (*SI Appendix*, Fig. S5 *J*–*L* and Datasets S1–S3). Respiration pathways were dramatically larger than glycolysis pathways in all three organisms (*SI Appendix*, Fig. S5 *J*–*L* and Datasets S1–S3). Strikingly, the increase in the size of respiration in relation to glycolysis is larger than the increase in yield afforded by respiration for all pathways except for fermentation in *E. coli* (*SI Appendix*, Fig. S5 *M*–*O*). Furthermore, 1.2 to 2.5-fold differences between the ratios of ATP yield to pathway size of glycolysis and respiration are similar to the respective differences in specific activity of ATP production (Fig. 2). These calculations further support our conclusion that the ATP production rate per mg of protein is higher for glycolysis than for respiration.

Our estimates for ATP yield ($\gamma_{\mathrm{glyc}}<\gamma_{\mathrm{resp}}$) and specific activity of ATP production ($V_{\max}^{\mathrm{glyc}}∙\gamma_{\mathrm{glyc}}>V_{\max}^{\mathrm{resp}}∙\gamma_{\mathrm{resp}}$) satisfied the parameter requirements in which our model predicts the switch from respiration to glycolysis at high glucose availability for the respiro-fermentative Pta-AckA pathway in *E. coli*, and redox-neutral fermentative glycolysis in *S. cerevisiae* and mammalian cells. If additional mitochondrial proteins are included in $\phi_{\mathrm{resp}}$, the specific activity of respiration would be even lower for *S. cerevisiae* and mammalian cells, reinforcing the robustness of our conclusion that glycolysis is faster than respiration per mg protein (*SI Appendix*, Fig. S8). Importantly, our results do not predict a benefit for switching from respiration to fermentative glycolysis in *E. coli*. Thus, in addition to explaining why *E. coli*, *S. cerevisiae,* and mammalian cells exhibit the Warburg Effect, our model provides an explanation for why *E. coli* exclusively use the respiro-fermentative Pta-AckA pathway in the presence of glucose and oxygen and not redox-neutral fermentative glycolysis as is the case for *S. cerevisiae*, and mammalian cells. Finally, we note that the specific activities of ATP production increase from mammalian cells to *E. coli* by over an order of magnitude for respiration and over fivefold for glycolysis, suggesting that these parameters can evolve and that there might be evolutionary pressure for microbes to increase the specific activity of ATP production.

### The Model with No Adjustable Parameters Quantitatively Predicts the Onset of Warburg Effect.

**Fig. 3.**
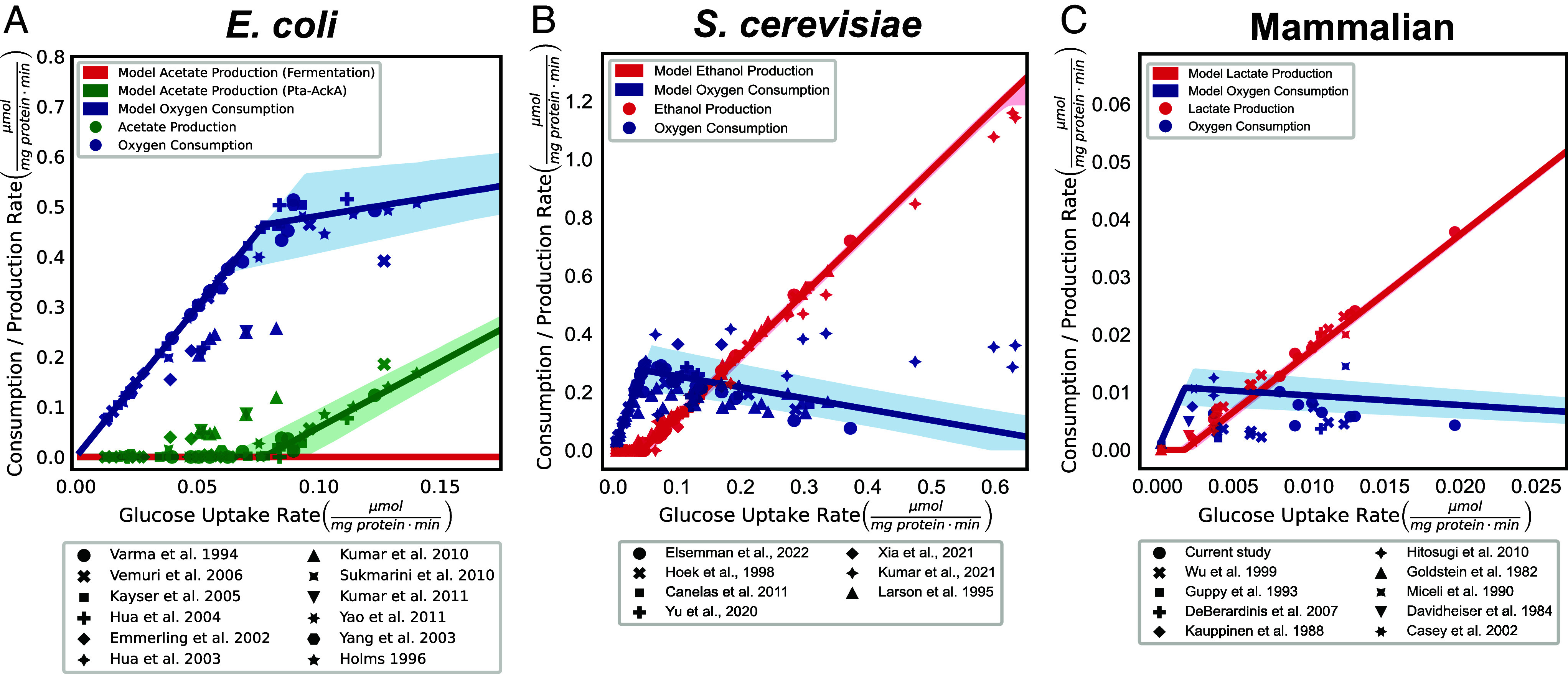
The model with no adjustable parameters that maximizes ATP production rate accurately predicts glycolysis and respiration rates and onset of the Warburg Effect in *E. coli*, *S. cerevisiae*, and mammalian cells. (*A*–*C*) Comparison of model predictions (lines) and experimental observations (points) for glycolysis (red) and respiration (blue) rates of *E. coli*, *S. cerevisiae*, and mammalian cells, respectively. Note that each unique point shape represents data from a distinct publication. The glucose uptake rate for each point is calculated from the sum of oxygen consumption and glycolytic by-product production.

Having demonstrated that our hypothesis predicts the benefit of the Warburg Effect, we next tested whether our model can quantitatively predict the onset of the Warburg Effect as well as rates of glycolysis and respiration measured under different experimental conditions in *E. coli*, *S. cerevisiae*, and mammalian cells. The additional biochemical parameter that we needed for this test was the total fraction of the proteome allocated to ATP-producing enzymes ($\phi_{\mathrm{total}}^{\mathrm{ATP}}$) that we estimated from proteomics data (*SI Appendix*, Fig. S5 *D*–*F* and Datasets S1–S3). We used glucose uptake rate as the only model input. For each given glucose uptake rate, the model used the four organism-specific biochemical parameters as described in the last section ($\gamma_{\mathrm{glyc}}$, $\gamma_{\mathrm{resp}}$, $V_{\max}^{\mathrm{glyc}}$, and $V_{\max}^{\mathrm{resp}}$) and the total fraction of the proteome allocated to ATP-producing enzymes ($\phi_{\mathrm{total}}^{\mathrm{ATP}}$) to determine the onset of the Warburg Effect as well as the glycolysis and respiration rates that allow cells to achieve a maximal ATP production rate (Fig. 3).

The datasets used for parameter estimation and model validation are entirely independent and differ qualitatively in several ways. Here, we detail how the datasets differ and how these datasets enable us to make quantitative predictions. To estimate model parameters, we use data collected under conditions that maximize either glycolysis or respiration rates. Conditions used to estimate glycolysis parameters include saturating glucose concentrations, respiratory inhibitors, or anaerobic cultures, while respiration parameters are estimated using nonfermentable substrates at saturating concentrations or ETC uncouplers. These experimentally derived parameter estimates enable our model to predict absolute rates of glycolysis and respiration rates depending on glucose uptake rate and proteome allocation to ATP-producing enzymes. We compared our predictions to independent data collected under aerobic conditions with variable glucose availability and no inhibitors or ETC uncouplers. Under these conditions, such as in a chemostat at low growth rates, glycolysis and respiration rates are typically much lower than the maximal rate determined by our parameter estimation. Therefore, the model output is not simply a fit of the data, but a demonstration of our model’s ability to predict independently generated data.

Our model accurately predicted the absolute rates of acetate, ethanol, and lactate production, and oxygen consumption over two orders of magnitude of glucose consumption rates in *E. coli*, *S. cerevisiae,* and mammalian cells (Fig. 3). Importantly, the results in Fig. 3 are not data fitting but predictions of the model using biochemical parameters estimated from independent experiments and no adjustable parameters. Most of the experimental data are within the 95% CI of model predictions. Our predications for *E. coli* indicate that the Pta-AckA pathway (Fig. 3*A*, green) is utilized for acetate production, while fermentation (Fig. 3*A*, red) is never utilized in the presence of oxygen (Fig. 3*A*). These model predictions align with our expectations based on the relative ATP yield per glucose and the specific activities of each pathway (Fig. 2*A*). Our predictions were robust to different methods of accounting either contribution of each pathway to biomass formation (*SI Appendix*, Fig. S9) in all three organisms or for mitochondrial proteome size in the estimation of $\phi_{\mathrm{total}}^{\mathrm{ATP}}$ and $V_{\max}^{\mathrm{resp}}$ for *S. cerevisiae* and mammalian cells (*SI Appendix*, Fig. S10). Finally, we note that we used average proteome allocation to ATP-producing enzymes across many conditions for each organism. This assumption is supported by our observation of the conservation of the proteome allocation to ATP-producing enzymes as compared to the protein translation space in response to changes in growth rate in glucose-limited chemostats in *E. coli* and *S. cerevisiae* or between mammalian cell lines (*SI Appendix*, Fig. S11). However, the size of ATP-producing space could change depending on cell type, nutrient availability, or disease state, so the accuracy of our model predictions could be further improved by measuring the proteome allocation to ATP-producing enzymes under relevant conditions.

### The Model Predicts the Onset of the Warburg Effect Independent of Growth Rate.

**Fig. 4.**
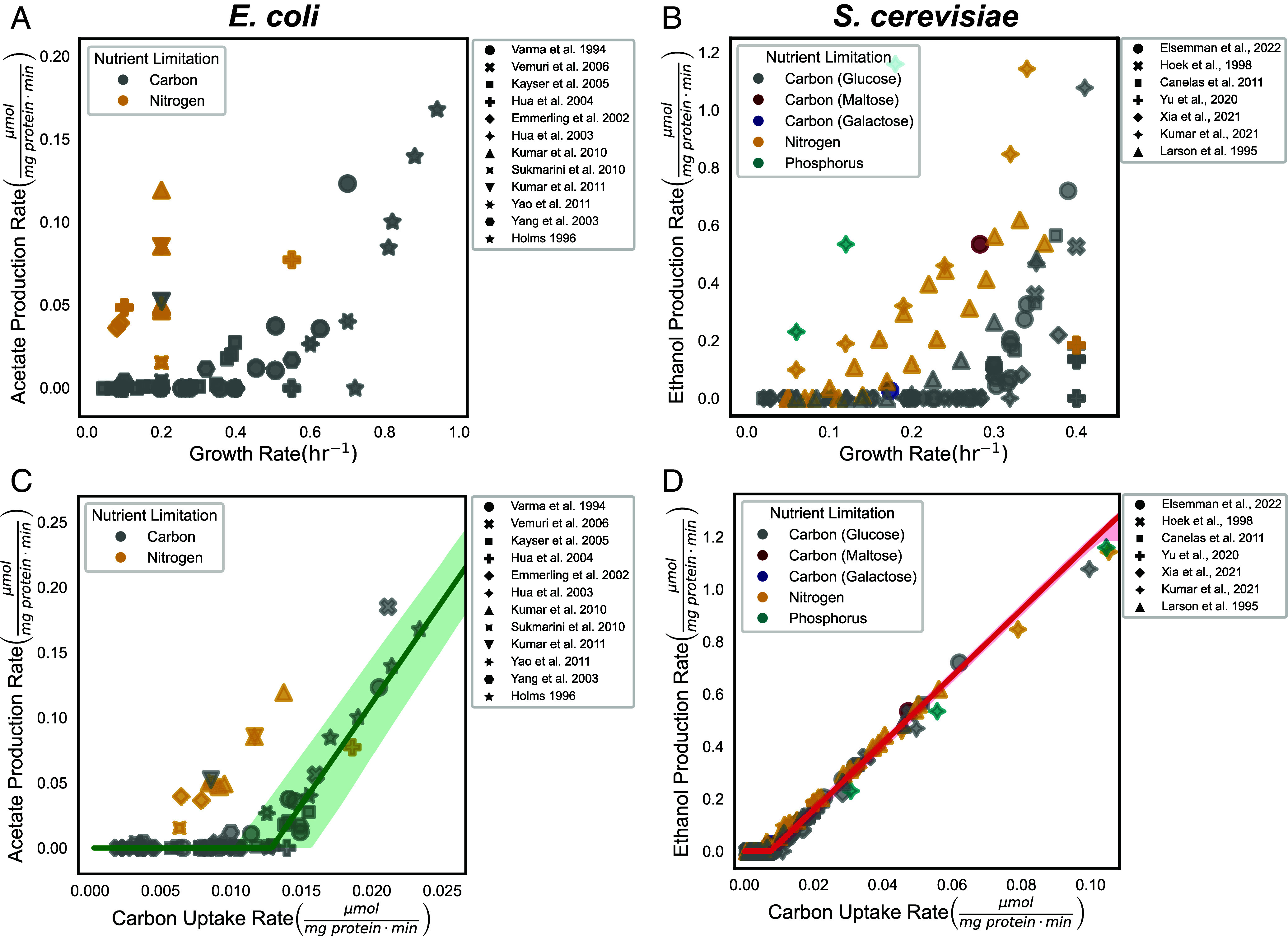
The Warburg Effect is driven by glucose availability and not growth rate. (*A*) Relationship between the growth rate (h^−1^) and the observed acetate production rate (μmol per mg cellular protein per min) for carbon- and nitrogen-limited cultures (gray and yellow, respectively) in *E. coli* or (*B*) the observed ethanol production rate (μmol per mg cellular protein per min) for carbon (glucose, maltose, galactose)-, nitrogen-, phosphorus-limited cultures (gray, burgundy, navy, yellow, green, respectively) in *S. cerevisiae*. (*C*) Relationship between the carbon uptake rate (μmol per mg cellular protein per min) and the observed acetate production rate (μmol per mg cellular protein per min) for carbon- and nitrogen-limited cultures (gray and yellow, respectively) in *E. coli* or (*D*) the observed ethanol production rate (μmol per mg cellular protein per min) for carbon (glucose, maltose, galactose)-, nitrogen-, phosphorus-limited cultures (gray, burgundy, navy, yellow, green, respectively) in *S. cerevisiae*. The carbon uptake rate for each point is calculated from the sum of oxygen consumption and glycolytic by-product production.

Our model proposes that glucose availability and not the growth rate is driving the onset of the Warburg Effect. To tease apart the role of growth rate and glucose availability, we have used *E. coli* and *S. cerevisiae* datasets from nitrogen, and phosphorus-limited growth conditions where carbon uptake rate and growth rate diverge significantly (Datasets S1–S3). In these datasets, the correlation between growth rate and glycolysis is weak (*ρ* = 0.50 and *ρ* = 0.43 for *E. coli* and *S. cerevisiae*, respectively) (Fig. 4 *A* and *B*) as compared to the correlation between the carbon uptake rate and glycolysis (*ρ* = 0.68 and *ρ* = 0.99, respectively), which has been previously observed for *S. cerevisiae* (39) (Fig. 4 *C* and *D*). Our model accurately predicted the relationship between the carbon uptake and glycolytic rates in *S. cerevisiae* irrespective of growth rate and nutrient limitation (Fig. 4*D*). Furthermore, our model can predict the onset of glycolysis for various carbon substrates, including glucose, maltose, and galactose without any alterations to the model as were done in a previous study to fit the data (38). For *E. coli*, our model overestimated the shift to acetate production under nitrogen-limited conditions (yellow points) (40). We speculate this is due to the known preference of *E. coli* to decrease the proteome fraction allocated to ATP-producing enzymes ($\phi_{\mathrm{total}}^{\mathrm{ATP}}$) at high glucose availability (22, 41). The latter challenges the constant $\phi_{\mathrm{total}}^{\mathrm{ATP}}$ assumption of our simple model under these specific conditions in *E. coli,* but is entirely consistent with our overall hypothesis that *E. coli* switched to the Pta-AckA glycolysis pathway because of its higher specific activity of ATP production in relation to respiration. We estimate that the proteome allocation dedicated to ATP-producing enzymes must undergo an approximate twofold decrease to account for the earlier onset of acetate production observed until nitrogen limitation (*SI Appendix*, Fig. S12). While the average proteome allocation to ATP-producing enzymes is 12%, we observed a range from as low as 6% to as high as 20% in our estimates of $\phi_{\mathrm{total}}^{\mathrm{ATP}}$ for *E. coli*. Therefore, twofold decrease in $\phi_{\mathrm{total}}^{\mathrm{ATP}}$ is within the observed range of the proteome allocation to ATP-producing enzymes. Furthermore, in cases in which the rate of change in the ATP-producing proteome space is known, our model is also able to predict resulting rates of glycolysis and respiration (see *SI Appendix* for details, *SI Appendix*, Fig. S13). These results suggest that under nitrogen- and phosphorus-limited conditions in *E. coli* and *S. cerevisiae*, glycolysis is utilized as a major ATP production strategy as compared to the glucose-limited culture with the same growth rate due to the excess availability of glucose (Fig. 4 *C* and *D*).

## Discussion

Our study tests a hypothesis that the Warburg Effect arises from the optimization of energy metabolism that allows cells to maximize the ATP production rate depending on glucose availability. We find that although cellular respiration generates more ATP per glucose molecule, the rate at which ATP is produced per the amount of enzyme protein involved is higher for the Pta-AckA pathway in *E. coli*, as well as for fermentative glycolysis in *S. cerevisiae*, and mammalian cells. Simply put, glycolysis is more compact than respiration, which allows glycolysis to produce ATP faster than respiration. Therefore, glycolysis is utilized when excess glucose is available and the ATP-producing space is limited. Our estimates show that in *E. coli*, only the Pta-AckA pathway and not redox-neutral fermentation is faster than respiration. Our observation explains a long-standing observation that *E. coli*, unlike mammalian cells or *S. cerevisiae*, do not use fermentation in the presence of oxygen (35).

We tested our hypothesis using a course-grained model of energy metabolism. Our model quantitatively predicts the onset of the Warburg Effect and glycolysis and respiration rates under diverse conditions in each of the three organisms using only the five biochemical parameters estimated with independent data, which provides strong support for our hypothesis. The ability of our model to make quantitative predictions provides a path for further validation of our hypothesis with additional experiments to improve estimates of the five model parameters and to generate more data connecting glucose uptake rate to glycolysis and respiration rates under different experimental conditions. Our model builds on the work of multiple groups that have utilized resource allocation models to explain the occurrence of the Warburg Effect in microbes (20–24). Our two contributions in terms of modeling are the use of measured biochemical parameters from independent experiments to quantitatively predict the occurrence of the Warburg Effect in each of the three organisms and the use of a resource allocation model to explain the occurrence of the Warburg Effect in mammalian cells.

To test our model, we have measured the specific activities of ATP production for both glycolysis and respiration. Our measurements for *E. coli* and *S. cerevisiae* complement previous estimates of these parameters (22, 23, 38). We note that the specific activity of the pathway has been referred to as “proteome efficiency” in several publications. Improved estimates of the specific activity of ATP production and measurement of specific activities of other pathways should allow the community to develop resource allocation models that predict cellular behavior beyond ATP production from independently measured biochemical parameters. Interestingly, we observed that the specific activities of ATP production for both glycolysis and respiration increase from mammalian cells through *S. cerevisiae* to *E. coli*, suggesting an evolutionary adaptation that may reflect selective pressure favoring more compact ATP synthetic pathways in microbes. An example of a molecular mechanism of such an adaptation is Complex I of the ETC. Mammalian proton-pumping Complex I has 45 subunits and weighs 1 MDa, while *E. coli* proton-pumping Complex I has only 13 subunits and weighs 0.5 MDa despite being homologous to the mammalian enzyme (42). In addition, both *E. coli* and *S. cerevisiae* have non-proton-pumping versions of Complex I (*S. cerevisiae* does not have a proton pumping Complex I at all) called NDH-II (43) and Ndi1 (44), respectively, that are not homologous to proton-pumping Complex I and have only one ~50 kDa subunit. Thus, the same or similar reactions are catalyzed by much smaller enzyme complexes in microbes than in mammalian cells, leading to higher specific activity of respiration in microbes. High specific activities may be selected for in microbial environments, such as soils, in which carbon substrates are typically scarce, but competition for them with other organisms is high. The lower rate of specific activities in mammalian cells may reflect the adaptation to multicellular cooperation, which represents investment in traits that are beneficial to the organism as a whole, but may come at the cost of vigor in an individual cell in the form of cellular function or metabolic activity (45, 46). Further investigation is necessary to determine the additional molecular mechanisms that drive the differences in the specific activity of ATP-producing pathways between species.

Our model could explain phenomena beyond the Warburg Effect or preference for the Pta-AckA pathway in rapidly growing cells. We show that the Warburg Effect depends on glucose availability rather than growth rate. Therefore, our model can be used to investigate the preference for glycolysis vs. respiration in differentiated cells. For example, fast-twitch muscle fibers are well known to prefer to use fermentation to produce energy used for short bursts of intensive physical activity (47). Fast-twitch muscle uses glycogen instead of glucose, which raises the yield of glycolysis from 2 to 3 ATP per glucose equivalent. Our estimates from Fig. 2*C*, in combination with the yield of 3 vs. 2 ATP per glucose, suggest that fast twitch muscle cells can produce ATP up to five times as fast if they dedicate the same fraction of their proteome to glycolysis than respiration–a large advantage in energy production rate that likely drove the evolution of fast-twitch muscle to rely on glycolytic fermentation. Moreover, our approach may clarify why microbes choose between expression of proton-pumping and non-proton-pumping versions of the same ETC complexes as mentioned above. Specifically, our model suggests that microbes could modulate the specific activity of their ATP-producing pathways by switching between high-yield (proton-pumping) and fast-rate (non-proton-pumping) respiratory chain components. This idea is supported by proteomics data showing that in *E. coli*, the expression of the faster, but lower-yielding non-proton-pumping NADH dehydrogenase II increases with growth rate, while expression of the high-yielding proton-pumping NADH dehydrogenase I decreases (48).

While our model provides a unified explanation for the Warburg Effect by focusing on ATP production rates, several limitations should be considered. First, our model only accounts for the occurrence of the Warburg Effect in three organisms, where our parameter estimates demonstrate that $\gamma_{\mathrm{glyc}}<\gamma_{\mathrm{resp}}$ and $V_{\max}^{\mathrm{glyc}}∙\gamma_{\mathrm{glyc}}>V_{\max}^{\mathrm{resp}}∙\gamma_{\mathrm{resp}}$. To robustly test our hypothesis, it would be especially valuable to measure our model parameters in organisms that do not exhibit the Warburg Effect, such as Crabtree-negative yeast. Specifically, we would aim to assess whether $\gamma_{\mathrm{glyc}}<\gamma_{\mathrm{resp}}$ and $V_{\max}^{\mathrm{glyc}}∙\gamma_{\mathrm{glyc}}<V_{\max}^{\mathrm{resp}}∙\gamma_{\mathrm{resp}}$ as would be predicted by our model. Second, the mathematical formulation of our model is intentionally simple, which inherently limits its ability to fully account for the complex and dynamic regulatory mechanisms that control respiratory and glycolytic rates. This limitation is evident by our observation that model parameters, such as fraction of the proteome occupied by ATP-producing enzymes, can vary under specific conditions like nitrogen limitation in *E. coli*. Our current model lacks the complexity to account for such dynamic changes. Additionally, our hypothetical model estimates for the regulation of glycolytic rates in response to oxygen limitation, have not experimentally tested where both may contribute to the high glycolytic rates observed in tumors with limited vasculature (49). Finally, we highlight that while our study provides an explanation for why it is beneficial for diverse organisms to switch between fast glycolysis and high-yielding respiration, more work needs to be done to investigate the molecular mechanisms of how cells optimize their energy metabolism, given the fundamental trade-offs of yield and rate.

## Materials and Methods

### Analysis Code and Figure Generation.

All data and code used in the figure generation are available as a GitHub repository via https://github.com/DenisTitovLab/WarburgEffectModel.

### Estimation of Proteome Occupancy by Metabolic Pathways ($\phi_{glyc}$, $\phi_{resp}$, and $\phi_{total}^{ATP}$).

We utilized a total protein approach and previously published measurements of the absolute quantification of proteins using mass spectrometry (MS) to estimate the fraction of the proteome occupied by glycolysis ()$\phi_{\mathrm{glyc}}$, respiration ()$\phi_{\mathrm{resp}}$, or all ATP-producing pathways ()$\phi_{\mathrm{total}}^{\mathrm{ATP}}$ (Datasets S1–S3). We only included studies that used 8 M urea or sodium dodecyl sulfate in the lysis buffer to improve the estimation of membrane proteins, which are not extracted using milder lysis buffers. The total protein approach assumes that a protein’s abundance within a cell’s proteome is proportional to that protein’s MS signal over the total MS signal (Eq. **7**).

[7] $$\frac{\mathrm{Protein}\;\mathrm{mass}}{\mathrm{Total}\;\mathrm{protein}\;\mathrm{mass}}\propto \frac{\mathrm{Protein}\;\mathrm{MS}\;\mathrm{Signal}}{\mathrm{Total}\;\mathrm{MS}\;\mathrm{Signal}}.$$

This approach was previously validated (50) by showing that it can accurately quantify proteins within their expected physiological range spanning five orders of magnitude.

ETC complexes are imbedded in the mitochondrial membrane and, therefore, are often extracted with less efficiency. The level of ETC complex subunits with greater than 20 percent missing values was imputed using the average levels of subunits of the same complex that where not missing on a per-sample basis. Both subunit mass and stoichiometry were accounted for in the correction.

The specific enzymes included in the calculation of $\phi_{\mathrm{glyc}}$ and $\phi_{\mathrm{resp}}$ for *E. coli*, *S. cerevisiae*, and mammalian cells are included in Datasets S1–S3. To match the respective physiological measurements for *E. coli* and *S. cerevisiae*, $\phi_{\mathrm{glyc}}$ was only calculated for fermentable carbon substrates (i.e., sugars) and $\phi_{\mathrm{resp}}$ is only calculated for nonfermentable carbon substrates (i.e., acetate, pyruvate, succinate, glycerol, fumarate) both in batch culture. For the respiro-fermentative Pta-AckA pathway in *E. coli*, ETC components were included to account for redox balance and additional ATP production. For the translational proteome space, the enzymes included in the Gene Ontology for translation (GO:0006412) were used for each organism.

All the estimates of respiration proteome occupancy reported in the main text included only core respiration and TCA cycle enzyme. To determine the effect of additional mitochondrial proteins on our predictions, we included two further predictions for *S. cerevisiae* and mammalian cells. First, in addition to core respiratory enzymes, we included mitochondrial proteins that positively correlated with the expression of the sum of the core respiration proteins (*ρ* > 0) and were statistically significant after Benjamini–Hochberg correction (*P* < 0.05). The statistical significance represents a two-tailed p-value, which is the probability of a correlation coefficient at least as big to be observed if the null hypothesis is true. All proteins included in the $\phi_{\mathrm{resp}}$ for *S. cerevisiae* and mammalian cells are listed in Datasets S2 and S3. Second, all MitoCarta mitochondrial proteins were included in the estimate $\phi_{\mathrm{resp}}$. The model estimations using these two additional estimates are reported in *SI Appendix*, Fig. S10.

For mammalian cells, 11 cell lines from distinct origins were utilized including A549, GAMG, HeLa, HepG2, Jurkat, K562, LnCap, MCF7, RKO, U2OS, and HEK293.

We did not correct proteome allocations for biosynthesis because the magnitude of the required correction is small (<30%, see discussion below) and accurate correction would require extensive metabolomics flux measurements that will complicate the model and discussion considerably. The magnitude of the correction is <30% for the following reasons. First, for respiration, the correction for biosynthesis flux will be small because a large fraction of the proteome allocated to respiration is occupied by respiratory chain which only carries energy production flux and not biosynthesis flux (e.g., see *SI Appendix*, Fig. S3). Furthermore, *E. coli* and *S. cerevisiae* grown on respiratory substrates divert a max of only ~45 to 55% of glucose to biosynthesis based on the biomass yield of ~0.45 to 0.55 g dry weight per g glucose. Second, the correction for glycolysis is small because cells grown in glucose in batch culture divert most of their glucose flux to glycolytic ATP production. For example, *E. coli* grown on glucose divert ~70% of glucose mass for acetate secretion (51) and the Pta-AckA pathway used under these conditions uses respiration so the correction is even smaller due to latter point. For *S. cerevisiae* grown on glucose ~80% of glucose mass is secreted as ethanol (52), and mammalian cells often use >90% of glucose mass for glycolytic fermentation as they are grown in rich media with amino acids and do not have to use as much glucose for biosynthesis. The correction of glycolysis for biosynthesis would have increased the specific activity of glycolysis making it even faster compared to respiration. We have now included the above description in the methods section.

### Estimation of Maximal Cellular Activity of Glycolysis and Respiration.

Absolute specific activities of glycolysis and respiration per weight of cellular proteins were estimated from experiments in which the maximal activity of each pathway was measured. These values are then combined with proteomic fractions of respective pathways to estimate specific activity per weight of pathway as describe in another section below. For *E. coli, S. cerevisiae*, and mammalian cells, the maximal acetate, ethanol, and lactate production rates (LPR), respectively, were used for glycolysis (Datasets S1–S3). For *E. coli and S. cerevisiae*, these values were taken from measurements grown in batch culture on a fermentable substrate. For mammalian cells, lactate production values were taken from measurements where cells were treated with oligomycin to maximize lactate production. For respiration, the maximal oxygen consumption rates (OCR) were utilized for *E. coli, S. cerevisiae*, and human cells (Datasets S1–S3). For *E. coli* and *S. cerevisiae*, these values were taken from measurements grown in batch culture or accelerostat on a nonfermentable substrate. For human cells, all oxygen consumption data are from Agilent Seahorse Bioanalyzer assays, and maximal respiration rate is estimated as the difference between oxygen consumption in the presence of uncoupler cyanide-p-trifluoromethoxyphenylhydrazone (FCCP) and respiration inhibitor Antimycin A (or Rotenone) to account for nonmitochondrial respiration. FCCP uncouples mitochondrial ETC activity from ATP production by dissipating the proton gradient, forcing the ETC to operate at a maximum rate, which allows the measurement of maximal OCR that is not limited by the ATP demand (53, 54). Each value was converted to units of μmol per mg cellular protein per min from oxygen consumption or glycolytic by-product formation to account only for glucose consumption toward ATP production and not biomass.

For studies that reported values in units consisting of gram cell dry weight (gCDW), it was calculated that 55% and 44% of dry cell mass consisted of protein in *E. coli* and *S. cerevisiae,* respectively (Datasets S1 and S2). The estimates of gCDW per g protein are based on the studies for *E. coli* and *S. cerevisiae* compiled in Datasets S1 and S2. For mammalian cells studies that reported values in units of cell number, representing 9 of 14 studies, it was assumed that each cell contained 300 pg of protein (50, 55, 56). The following mammalian cell lines were utilized for these estimates: C2C12, H1299, HeLa, 4T1, K562, CD4^+^ T cell, primary isolated cardiomyocytes, Ehrlich ascites tumor cells, and primary rat cerebellar neurons.

### Estimation of the Molecular Weight of Glycolysis and Respiration Pathways.

Molecular weights of the pathways were calculated by summing the molecular weights of all proteins in each of the pathways corrected for pathway stoichiometry (Datasets S1–S3). For each enzyme that has isoforms, the average molecular weight of isoforms was used. For each enzyme that is a multisubunit protein complex, the sum of the subunits was used with subunit stoichiometry accounted.

### Estimation of ATP Yield Per Glucose of Glycolysis and Respiration.

The ATP yield for (*γ_resp_* and *γ_glyc_*) was estimated for *E. coli*, *S. cerevisiae*, and mammalian cells. Fermentative glycolysis yield of two ATP per glucose is well defined for all three organisms (*SI Appendix*, Fig. S4 *A*–*C* and Datasets S1–S3).

To define the ATP yield of respiration for each organism, experimentally derived measurements (57–59), in combination with the following calculations, were used to account for ATP produced from glycolysis, the TCA cycle, and the ETC (Datasets S1–S3). In fermentative glycolysis, glucose is converted to a glycolytic by-product, producing a net gain of two ATP per glucose for each organism.

Fermentative glycolysis for *E. coli*, *S. cerevisiae*, and mammalian cells, respectively:

[8] $$C_{6}H_{12}O_{6}+2\;\text{ADP}+2\;\text{Pi}\to \text{Various}\;\text{Products}+2\;\text{ATP},$$

[9] $$C_{6}H_{12}O_{6}+2\;\mathrm{ADP}+2\;\mathrm{Pi}\to 2C_{6}H_{12}O+2{\mathrm{CO}}_{2}+2\;\mathrm{ATP},$$

[10] $$C_{6}H_{12}O_{6}+2\;\mathrm{ADP}+2\;\mathrm{Pi}\to 2C_{3}H_{6}O_{3}+2\;\mathrm{ATP}.$$

In respiration, glucose is converted to a pyruvate, generating ATP and NADH. Pyruvate is further oxidized through the TCA cycle, generating additional NADH, CoQH_2_, and ATP per glucose in all three organisms.

[11] $$\begin{array}{c} C_{6}H_{12}O_{6}+6O_{2}+4\;\mathrm{ADP}+4\;\mathrm{Pi}+2\;\mathrm{CoQ}+10\;{\mathrm{NAD}}^{+}\\ +14\;H^{+}\to 6\;{\mathrm{CO}}_{2}+6H_{2}O+4\;\mathrm{ATP}+2\;{\mathrm{CoQH}}_{2}+10\;\mathrm{NADH}. \end{array}$$

In the ETC, NADH and FADH_2_ are oxidized to generate a proton gradient in the inner mitochondrial membrane (or plasma membrane in *E. coli*). In *S. cerevisiae* and mammalian cells, the NADH produced from glycolysis is shuttled into the mitochondria to enter the ETC at a cost of 1 H^+^ per NADH. The proton gradient is then used to drive the production of ATP synthase. Each organism has a unique number of H^+^ translocated per NADH and FADH_2_ based on the translocated H^+^ per electron ratio of the utilized ETC components. For *E. coli*, the ratio of translocated H^+^ per electron is 2 for NADH-ubiquinone reductase (NDH-I) and 2 for quinol oxidase (cytochrome bo_3_ oxidase). For *S. cerevisiae*, the ratio of translocated H^+^ per electron is 0 for NADH-ubiquinone reductase (Ndi1), 1 for quinol-cytochrome c reductase (Complex III), and 2 for cytochrome c oxidase (Complex IV). For mammalian cells, the ratio of translocated H^+^ per electron is 2 NADH-ubiquinone reductase (Complex I), 1 for quinol-cytochrome c reductase (Complex III), and 2 for cytochrome c oxidase (Complex IV). Note that each NADH or FADH_2_ donates two electrons. Therefore, the total number of translocated H^+^ is calculated by the following equation where mammalian cells are used as an example:

[12] $$\begin{array}{c} H_{\text{maximum}}^{+}=2∙H_{\text{CI}}^{+}∙{\text{NADH}}_{\text{total}}+2∙H_{\text{CIII}}^{+}∙({\text{NADH}}_{\text{total}}+{\mathrm{FADH}}_{2_{total}})\\ +\;2∙H_{\text{CVI}}^{+}∙(\mathrm{NADH}+{\mathrm{FADH}}_{2})-{\text{NADH}}_{\text{glycolysis}}∙H_{\text{Transport}}^{+}. \end{array}$$

However, it is well known that proton leak occurs in the membrane. For mammalian cells, the proton leak was calculated from the difference in the oxygen consumption rate in the presence of oligomycin and antimycin A (or rotenone) (Dataset S3). We determined that the proton leak was ~25%. For *E. coli* and *S. cerevisiae*, we also assumed ~75% of theoretical maximal yield to account for proton leak. Furthermore, the ratio of H^+^/ATP was measured to be 4, 3, and 3 for *E. coli*, *S. cerevisiae*, and mammalian cells, respectively (57–59). Note that in mammalian cells and *S. cerevisiae*, we assume the adenine nucleotide translocator (ANT) is active, and therefore, the cost of transporting each ATP from the mitochondria is 1 H^+^. Therefore, the total number of ATP produced through respiration is calculated by

[13] $$\gamma_{\mathrm{resp}}=\frac{\left(H_{\text{maximum}}^{+}-H_{\text{Leak}}^{+}\right)}{\left(H^{+}/\mathrm{ATP}+H^{+}/{\mathrm{ATP}}_{\mathrm{transport}}\right)}.$$

The ATP yield of respiration was determined to be 20, 16, and 24 ATP per molecule of glucose for *E. coli*, *S. cerevisiae*, and mammalian cells, respectively:

[14] $$C_{6}H_{12}O_{6}+6\;O_{2}+20\;\mathrm{ADP}+20\;\mathrm{Pi}\to 6\;{\mathrm{CO}}_{2}+6\;H_{2}O+20\;\mathrm{ATP},$$

[15] $$C_{6}H_{12}O_{6}+6\;O_{2}+16\;\mathrm{ADP}+16\;\mathrm{Pi}\to 6\;{\mathrm{CO}}_{2}+6\;H_{2}O+16\;\mathrm{ATP},$$

[16] $$C_{6}H_{12}O_{6}+6\;O_{2}+24\;\mathrm{ADP}+24\;\mathrm{Pi}\to 6\;{\mathrm{CO}}_{2}+6\;H_{2}O+24\;\mathrm{ATP}.$$

The Pta-AckA pathway in *E. coli* utilizes the ETC for oxidation of the 4 NADH produced per molecule of glucose in the *E. coli* Pta-AckA pathway that converts glucose to acetate, yielding 10 ATP per molecule of glucose. The Pta-AckA pathway in *E. coli* utilizes the ETC for oxidation of the 4 NADH produced per molecule of glucose in the *E. coli* Pta-AckA pathway that converts glucose to acetate.

[17] $$\begin{array}{c} C_{6}H_{12}O_{6}+2\;O_{2}+4\;\mathrm{ADP}+4\;\mathrm{Pi}+4\;{\mathrm{NAD}}^{+}\\ +\;4\;H^{+}\to 2\;C_{2}H_{4}O_{2}+2\;{\mathrm{CO}}_{2}+2\;H_{2}+2\;H_{2}O\;+4\;\mathrm{ATP}+4\;\mathrm{NADH}. \end{array}$$

### Estimation of the Specific Activity of ATP Production of Glycolysis and Respiration.

We used experimental data to estimate the specific activities of ATP production of glycolysis and respiration (*SI Appendix*, Fig. S4 *G*–*I* and Datasets S1–S3). The specific activity of glucose consumption was calculated by taking the maximal cellular glucose consumption rate by a given pathway (µmol glucose per mg of cellular protein per min) and dividing it by the fraction of the proteome occupied by the pathway ($\phi_{\mathrm{glyc}}$ or $\phi_{\mathrm{resp}}$).

[18] $$V_{\max}^{glyc/resp}=\frac{{Max\;cellular\;rate}_{glyc/resp}}{\phi_{glyc/resp}}.$$

As described above in *Estimation of Maximal Cellular Activity of Glycolysis and Respiration*, we determined the maximal glycolysis and respiration rates for *E. coli, S. cerevisiae*, and mammalian cells by compiling an extensive dataset from 38 independent publications containing 57 measurements of production rate of glycolysis products (i.e., acetate, ethanol, lactate) and OCR, which were converted to glucose consumption rate using known stoichiometry of the pathways (Datasets S1–S3). For each organism, we calculated $\phi_{\mathrm{glyc}}$_,_ and $\phi_{\mathrm{resp}}$ from proteomics data as described above in *Estimation of Proteome Occupancy by Metabolic Pathways* $\left(\phi_{\mathrm{glyc}},\;\phi_{\mathrm{resp}},\;\text{and}\;\phi_{\mathrm{total}}^{\mathrm{ATP}}\right)$.

We made two additional predictions for *S. cerevisiae*, and mammalian cells that were only used for data reported in *SI Appendix*, Figs. S6 and S7. In the first, corrections to $\phi_{\mathrm{resp}}$ to account for mitochondrial proteins that are required for mitochondrial biogenesis and function but are not core respiration components by including into $\phi_{\mathrm{resp}}$ mitochondrial proteins whose expression was significantly (*P* < 0.05) and positively correlated (*ρ > 0*) with the sum of the TCA and ETC proteins. In the second, we included all mitochondrial proteins.

### Mathematical Model.

To describe the Warburg Effect, we use a constrained optimization model. The objective function of the model is to maximize the ATP production rate given the constraints as described in Eqs. **19**–**21** reproduced here for convenience:

[19] $$Maximize:\;V_{\mathrm{ATP}}=V_{\mathrm{glyc}}∙\gamma_{\mathrm{glyc}}+V_{\mathrm{resp}}∙\gamma_{\mathrm{resp}}.$$

[20] $$Subject\;to:\frac{V_{\mathrm{glyc}}}{V_{\max}^{\mathrm{glyc}}}+\frac{V_{\mathrm{resp}}}{V_{\max}^{\mathrm{resp}}}\leq \phi_{\mathrm{total}}^{\mathrm{ATP}},$$

[21] $$V_{\mathrm{glyc}}+V_{\mathrm{resp}}\leq V_{\mathrm{glucose}}.$$

We can use the $V_{\mathrm{glyc}}$ and $V_{\mathrm{resp}}$ values to calculate the absolute rates of glycolytic by-product (i.e., acetate, ethanol, or lactate) or oxygen consumption from the predicted rates of glycolysis and respiration (Eqs. **22** and **23**, respectively):

[22] $$V_{\mathrm{glyc}}^{\mathrm{byproduct}}=\gamma_{\mathrm{gluc}}^{\mathrm{byproduct}}∙V_{\mathrm{glyc}},$$

[23] $$V_{\mathrm{resp}}^{O2}=\gamma_{\mathrm{resp}}^{O2,consumed}∙V_{\mathrm{resp}}.$$

We used the method of Lagrange multipliers with Karush-Kuhn-Tucker conditions to solve this optimization problem with inequality constraints. We report an analytical solution here. We note that the global optimum can also be found using the linear programming software PuLP (60). The full derivation is presented in *SI Appendix, Supplemental Discussion 1*. Here, we present the full set of cases, unlike in the main text where we have just provided those which have biological relevance. With $V_{\max}^{\mathrm{glyc}},\gamma_{\mathrm{glyc}},V_{\max}^{\mathrm{resp}},\gamma_{\mathrm{resp}},\phi_{\mathrm{total}}^{\mathrm{ATP}},V_{\mathrm{glucose}}>0,$ the solution is explicitly written by cases:

[24] $$\begin{array}{c} \text{If}\begin{array}{c} \gamma_{glyc}<\;\gamma_{resp}\\ V_{max}^{resp}∙\phi_{total}^{ATP}>\;V_{glucose} \end{array}\;\text{then}\;V_{glyc}=0,\;V_{resp}=V_{glucose}. \end{array}$$

[25] $$\begin{array}{c} \mathrm{If}\;\begin{array}{c} \;\gamma_{\mathrm{glyc}}<\gamma_{\mathrm{resp}}\\ V_{\max}^{\mathrm{glyc}}∙\gamma_{\mathrm{glyc}}>V_{\max}^{\mathrm{resp}}∙\gamma_{\mathrm{resp}}\\ \;\phi_{\mathrm{total}}^{\mathrm{ATP}}∙V_{\max}^{\mathrm{glyc}}>V_{\mathrm{glucose}}>\phi_{\mathrm{total}}^{\mathrm{ATP}}∙V_{\max}^{\mathrm{resp}} \end{array}\\ \mathrm{then}\;\begin{array}{c} V_{\mathrm{glyc}}=\frac{\phi_{\mathrm{total}}^{\mathrm{ATP}}∙V_{\max}^{\mathrm{glyc}}∙V_{\max}^{\mathrm{resp}}-V_{\mathrm{glucose}}∙V_{\max}^{\mathrm{glyc}}}{V_{\max}^{\mathrm{resp}}-V_{\max}^{\mathrm{glyc}}}\\ V_{\mathrm{resp}}=\frac{V_{\max}^{\mathrm{resp}}(\phi_{\mathrm{total}}^{\mathrm{ATP}}∙V_{\max}^{\mathrm{glyc}}∙V_{\max}^{\mathrm{resp}}-V_{\mathrm{glucose}})}{V_{\max}^{\mathrm{glyc}}-V_{\max}^{\mathrm{resp}}} \end{array}. \end{array}$$

[26] $$\begin{array}{c} If\;\begin{array}{c} V_{\max}^{\mathrm{glyc}}∙\gamma_{\mathrm{glyc}}>V_{\max}^{\mathrm{resp}}∙\gamma_{\mathrm{resp}}\\ V_{\max}^{\mathrm{glyc}}∙\phi_{\mathrm{total}}^{\mathrm{ATP}}<V_{\mathrm{glucose}} \end{array}\;\text{then}\;V_{\mathrm{glyc}}=V_{\max}^{\mathrm{glyc}}\cdot \phi_{\mathrm{total}}^{\mathrm{ATP}},V_{\mathrm{resp}}=0. \end{array}$$

[27] $$\begin{array}{c} If\;\begin{array}{c} \gamma_{\mathrm{glyc}}>\gamma_{\mathrm{resp}}\\ V_{\max}^{\mathrm{glyc}}∙\phi_{\mathrm{total}}^{\mathrm{ATP}}>V_{\mathrm{glucose}} \end{array}\;\mathrm{then}\;V_{\mathrm{glyc}}=V_{\mathrm{glucose}},V_{\mathrm{resp}}=0. \end{array}$$

[28] $$\begin{array}{c} \mathrm{If}\;\begin{array}{c} \gamma_{\mathrm{glyc}}>\gamma_{\mathrm{resp}}\\ V_{\max}^{\mathrm{glyc}}∙\gamma_{\mathrm{glyc}}<V_{\max}^{\mathrm{resp}}∙\gamma_{\mathrm{resp}}\\ \phi_{\mathrm{total}}^{\mathrm{ATP}}∙V_{\max}^{\mathrm{glyc}}<V_{\mathrm{glucose}}<\phi_{\mathrm{total}}^{\mathrm{ATP}}∙V_{\max}^{\mathrm{resp}} \end{array}\\ \mathrm{then}\;\begin{array}{c} V_{\mathrm{glyc}}=\frac{\phi_{\mathrm{total}}^{\mathrm{ATP}}∙V_{\max}^{\mathrm{glyc}}∙V_{\max}^{\mathrm{resp}}-V_{\mathrm{glucose}}∙V_{\max}^{\mathrm{glyc}}}{V_{\max}^{\mathrm{resp}}-V_{\max}^{\mathrm{glyc}}}\\ V_{\mathrm{resp}}=\frac{V_{\max}^{\mathrm{resp}}(\phi_{\mathrm{total}}^{\mathrm{ATP}}∙V_{\max}^{\mathrm{glyc}}∙V_{\max}^{\mathrm{resp}}-V_{\mathrm{glucose}})}{V_{\max}^{\mathrm{glyc}}-V_{\max}^{\mathrm{resp}}} \end{array}. \end{array}$$

[29] $$\begin{array}{c} \text{If}\;\begin{array}{c} V_{\mathrm{glyc}}∙\gamma_{\mathrm{glyc}}<V_{\mathrm{resp}}∙\gamma_{\mathrm{resp}}\\ V_{\max}^{\mathrm{resp}}∙\phi_{total}^{\mathrm{ATP}}\;\leq \;V_{\mathrm{glucose}} \end{array}\text{then}\;V_{\mathrm{glyc}}=0,V_{\mathrm{resp}}=V_{\max}^{\mathrm{resp}}∙\phi_{\mathrm{total}}^{\mathrm{ATP}}. \end{array}$$

To predict the absolute rates of three pathways in *E. coli*, fermentation, Pta-AckA, and respiration, we extended the model to include the Pta-AckA pathway as follows:

[30] $$Maximize:V_{\mathrm{ATP}}=V_{\mathrm{ferm}}∙\gamma_{\mathrm{ferm}}+V_{\mathrm{pta}}∙\gamma_{\mathrm{pta}}+V_{\mathrm{resp}}∙\gamma_{\mathrm{resp}}.$$

[31] $$Subject\;to:\frac{V_{\mathrm{ferm}}}{V_{\max}^{\mathrm{ferm}}}+\frac{V_{\mathrm{pta}}}{V_{\max}^{\mathrm{pta}}}+\frac{V_{\mathrm{resp}}}{V_{\max}^{\mathrm{resp}}}\leq \phi_{\mathrm{total}}^{\mathrm{ATP}},$$

[32] $$V_{\mathrm{ferm}}+V_{\mathrm{pta}}+V_{\mathrm{resp}}\leq V_{\mathrm{glucose}}.$$

We used the method of Lagrange multipliers with Karush-Kuhn-Tucker conditions to solve this optimization problem with inequality constraints for the additional Pta-AckA pathway. Since adding a third pathway increases the number of cases for the analytical solution, we also present the three relevant cases to demonstrate that given the relationship between the yield (i.e., $\gamma_{\mathrm{ferm}}<\gamma_{\mathrm{pta}}<\gamma_{\mathrm{resp}}$) and the specific activity (i.e., $\gamma_{\mathrm{ferm}}<\gamma_{\mathrm{resp}}<\gamma_{\mathrm{pta}}$) of the three pathways (Fig. 2*A* and *SI Appendix*, Fig. S4*A*, respectively). We limited our analysis to demonstrate that fermentation would never be under the estimated parameter relationships (*SI Appendix, Supplementary Discussion 1*). The following three cases describe that fermentation is not an energetically favorable option with oxygen is available:

[33] $$\begin{array}{c} If\;\begin{array}{c} \;\gamma_{ferm}<\;\gamma_{resp}\\ \gamma_{pta}<\;\gamma_{resp}\\ \;V_{max}^{resp}∙\phi_{total}^{ATP}>\;V_{glucose} \end{array}\;\text{then}\;V_{ferm}=0,\;V_{pta}=0,\;V_{resp}=V_{glucose}. \end{array}$$

[34] $$\begin{array}{c} \begin{array}{cc} If & \begin{array}{c} \gamma_{\mathrm{ferm}}<\gamma_{\mathrm{resp}}\\ \gamma_{\mathrm{pta}}<\gamma_{\mathrm{resp}}\\ V_{\max}^{\mathrm{ferm}}∙\gamma_{\mathrm{ferm}}<V_{\max}^{\mathrm{pta}}∙\gamma_{\mathrm{pta}}\\ V_{\max}^{\mathrm{pta}}∙\gamma_{\mathrm{pta}}>V_{\max}^{\mathrm{resp}}∙\gamma_{\mathrm{resp}}\\ \phi_{\mathrm{total}}^{\mathrm{ATP}}∙V_{\max}^{\mathrm{glyc}}>V_{\mathrm{glucose}}>\phi_{\mathrm{total}}^{\mathrm{ATP}}∙V_{\max}^{\mathrm{resp}} \end{array}\\ \mathrm{then}\; & \begin{array}{c} V_{\mathrm{ferm}}=0\\ V_{\mathrm{pta}}=\frac{\phi_{\mathrm{total}}^{\mathrm{ATP}}∙V_{\max}^{\mathrm{pta}}∙V_{\max}^{\mathrm{resp}}-V_{\mathrm{glucose}}∙V_{\max}^{\mathrm{pta}}}{V_{\max}^{\mathrm{resp}}-V_{\max}^{\mathrm{pta}}}\\ V_{\mathrm{resp}}=\frac{V_{\max}^{\mathrm{resp}}(\phi_{\mathrm{total}}^{\mathrm{ATP}}∙V_{\max}^{\mathrm{pta}}∙V_{\max}^{\mathrm{resp}}-V_{\mathrm{glucose}})}{V_{\max}^{\mathrm{pta}}-V_{\max}^{\mathrm{resp}}} \end{array}\;. \end{array} \end{array}$$

[35] $$\begin{array}{c} \begin{array}{cc} If & \begin{array}{c} V_{\max}^{\mathrm{ferm}}∙\gamma_{\mathrm{ferm}}<V_{\max}^{\mathrm{pta}}∙\gamma_{\mathrm{pta}}\\ V_{\max}^{\mathrm{pta}}∙\gamma_{\mathrm{pta}}>>V_{\max}^{\mathrm{resp}}∙\gamma_{\mathrm{resp}}\\ V_{\max}^{\mathrm{pta}}∙\phi_{\mathrm{total}}^{\mathrm{ATP}}<V_{\mathrm{glucose}} \end{array}\mathrm{then}\;V_{\mathrm{ferm}}=0,V_{\mathrm{pta}}=V_{\max}^{\mathrm{glyc}}∙\phi_{\mathrm{total}}^{\mathrm{ATP}},V_{\mathrm{resp}}=0. \end{array} \end{array}$$

To estimate the CI of model prediction, we performed bootstrapping to resample the five parameter estimates (i.e., fraction of the proteome occupied by pathway, specific activity, and ATP yield) with replacement (N = 10,000). After each round of sampling, the specific activity of each pathway and total proteome occupancy of ATP-production enzymes is calculated as described previously. The sampled parameter values for proteome occupied by pathway, specific activity, and ATP yield from each round of bootstrapping were then used for either the linear program or analytical solution described above. All 10,000 results were stored then the mean was plotted with the 95% CI, signifying the confidence levels. For *S. cerevisiae* and mammalian cells, two additional estimates were conducted 1) with correlated additional mitochondrial proteins and 2) with all mitochondrial proteins (*SI Appendix*, Fig. S8 and Datasets S2 and S3).

To ensure that we accurately model only the metabolic fluxes involved in energy production and not biomass production, we calculated the glucose uptake rate by applying the stoichiometric ratios derived from oxygen consumption and glycolytic by-product formation. The glucose uptake rate used for energy production was then determined by the following:

[36] $$Glucose\;Uptake\;Rate=\frac{V_{\mathrm{resp}}^{O_{2}\;consumption}}{6}+\frac{V_{\mathrm{glyc}}^{by-product\;\mathrm{production}}}{2}.$$

### Cell Lines and Cell Culture.

All cell lines were cultured in Dulbecco’s modified eagle’s medium [DMEM (Gibco™ 12800082), 3.7 g/L NaHCO_3_, 10% FBS (Gibco™ 10437028), and 100 U/mL penicillin–streptomycin (Gibco™ 15140122)]. All experiments were performed in the absence of penicillin–streptomycin. The following cell lines were used: C2C12, Huh7, HeLa, U2OS, A549, BR3, H1703, PC3, and MCF7. In addition, published data from following cell lines were used to compare our model estimates: SiHa, Bcap37, 4T1, H1299, HepG2, SW620, HeLa, SKBR3, A549, SF188, primary thymocytes from Wistar rat, primary astrocytes from Wistar rat, H1299, human fibroblast, human retinal pigment epithelium, Anococcygeus muscle from Wistar rat, and primary cardiomyocytes from Sprague-Dawley rats.

### Measurement of Cell Volume.

During each cell passage, the Coulter Z2 Counter Cell Particle Analyzer (Beckman) was used to determine the concentration and volume of cells. The average volume (fL) and SD of each cell line are reported in Dataset S3.

### Cell Protein Concentration Measurement.

Protein concentration was determined using the bicinchoninic acid (BCA) assay. One million cells were collected in a 2 mL centrifuge tube. Cells were pelleted at 600 g. Media were aspirated. Cells were washed with phosphate-buffered saline, spun, and aspirated twice to remove protein contained in the media. Cells were resuspended in 500 µL of lysis buffer containing 1% sodium dodecyl sulfate and incubated at 90 °C for 10 min. Samples were prepared for analysis in triplicate as instructed in the Pierce™ BCA Protein Assay Reagent Kit (Thermo Fisher Scientific 23225). Samples intensity was measured at 562 nm using BioTek Cytation1. Protein concentrations per cell volume for each analyzed mammalian cell line as compared to a bovine serum albumin standard (Dataset S3).

### Measurement of LPR.

Lactate Production Rate (LPR) of cell lines were measured using the L-Lactate Assay Kit-I (Colorimetric) (Eton Bioscience 120001400A). Cells were seeded at the volumetric equivalent of 0.5 million HeLa cells, where the average HeLa cell volume was measured to be 2,300 fL, per well of a 6-well plate in 3 mL of DMEM and were incubated at 37 °C in 5% CO2 incubator. The medium was replaced 24 h later with 3 mL of the assay medium [DMEM (Gibco™ 12100061), 10 % dialyzed FBS (Gibco™ 26400044) and 3.7 g/L NaHCO_3_]. Two hundred µL of media were collected every hour for 4 h and immediately frozen on dry ice. Samples were stored at −80 °C until analyzed. At the end of the 4-h sampling period, cells were trypsinized and counted using the coulter counter. To analyze the samples, 50 µL of sample was combined with 50 µL L-Lactate assay solution. The absorbance at 490 nm was measured for 45 min at 37 °C on the BioTek Cytation1. The slope of each well was determined. A standard curve was made by plotting the slope of OD490 nm values for each L-Lactate standard as a function of L-Lactate concentration. The L-Lactate concentration of each biological sample was determined using the equation obtained from the linear regression of the standard curve. L-Lactate in each sample was converted from concentration to moles per g protein using obtained cell counts and protein concentrations per cell volume for each mammalian cell line. Linear regression was performed across the 4-h time course to determine the LPR for each cell line.

### Measurement of Oxygen Consumption Rate.

Oxygen Consumption Rate (OCR) of cell lines were measured with the Agilent Seahorse XFe24 Analyzer. Cells were seeded at the volumetric equivalent of 100,000 HeLa cells, where the average HeLa cell volume was measured to be 2,300 fL, per well of XFe24 cell culture microplates in 150 µL of DMEM, and were incubated at 37 °C in 5% CO_2_ incubator. The medium was replaced 24 h later with 500 µL of the assay medium [DMEM (Gibco™ 12100061), 10% dialyzed FBS (Gibco™ 10437028), and 5 mM HEPES-KOH, pH 7.4], and the plates were placed in the Agilent Seahorse XFe24 Analyzer for OCR measurements. Each measurement was performed over 4 min after a 2 min mix and 2 min wait period. Basal measurements were collected three times, three measurements were collected after injection of oligomycin (final concentration of 1 µM), three measurements were collected after injection of FCCP (final concentration of 3 µM), three measurements were collected after a subsequent addition of FCCP (final concentration of 6 µM), three measurements were collected after addition of antimycin A and rotenone (final concentration of 1 µM each). Each drug was injected as a concentrated 50 µL solution of the assay medium. At the end of the measurements, cells were trypsinized and counted using the Coulter Z2 Counter Cell Particle Analyzer (Beckman). OCR were converted to µmol per mg protein per min using obtained cell counts and protein concentrations per cell volume for each mammalian cell line.

## Supplementary Material

Appendix 01 (PDF)

Dataset S01 (XLSX)

Dataset S02 (XLSX)

Dataset S03 (XLSX)

## Data Availability

Code used in the figure generation is available as a GitHub repository via https://github.com/DenisTitovLab/WarburgEffectModel (61). All data are available in the main text or supporting information.
