# Supplementary material for: The Warburg Effect is the result of faster ATP production by glycolysis than respiration
Source: Proc Natl Acad Sci U S A. 2024 Nov 8;121(46):e2409509121. doi: 10.1073/pnas.2409509121 (PMC11573683; doi:10.1073/pnas.2409509121)
Supplement: Supplementary file 1 — Appendix 01 (PDF) [file pnas.2409509121.sapp.pdf]

Supporting Information for
The Warburg Effect is the result of faster ATP production by glycolysis than respiration

Matthew A. Kukurugya[1,3], Saharon Rosset[4], Denis V. Titov[1,2,3,*]

[1]Department of Molecular & Cell Biology, University of California; Berkeley CA, 94720
[2]Department of Nutritional Sciences & Toxicology, University of California; Berkeley CA, 94720
[3]Center for Computational Biology, University of California; Berkeley CA, 94720
[4]Department of Statistics and Operations Research, Tel Aviv University; Tel Aviv, Israel, 69978

*Denis V. Titov
Email: titov@berkeley.edu

This PDF file includes:

Supporting text
Figures S1 to S13

Supplementary Discussion 1

Overview of Linear Program and Derivation of Analytical Solution

Here we present our model of cellular ATP production. We aimed to maintain a simple mathematical model to allow for intuitive biological interpretation of both the underlying framework as well as the model outputs. Therefore, the model uses five biochemical parameters, including the ATP yield (γ_{glyc} and γ_{resp}) and specific activity (V_{glyc} and V_{resp}) of glycolysis and respiration and fraction of the proteome that can be occupied by ATP-producing enzymes (ϕ_{total}^{ATP}).

Model parameters:

- V_{total}^{ATP} represent the total ATP production rate (μmol mg protein^{-1} min^{-1})
- V_{max}^{glyc} and V_{max}^{resp} represent the specific activity of pathway (μmol mg pathway^{-1} min^{-1})
- γ_{glyc} and γ_{resp}represent the yield of ATP per glucose for the pathway (unitless)
- ϕ_{total}^{ATP} represent the fraction of the proteome dedicated to ATP-producing enzymes

The purpose of the model is to pointedly test our hypothesis that the Warburg Effect allows cells to produce ATP at a maximal rate. Accordingly, we chose to use linear programming, a mathematical method for optimizing a linear objective function subject to given constraints. The goal of the linear program is to find values of the free variable that maximize or minimize the objective function while satisfying the constraints. Our objective function is written to maximize the ATP production rate (V_{total}^{ATP}) (equation 1) by changing the rates V_{glyc} and V_{resp} within the allocated total proteome space for ATP-producing enzymes (ϕ_{total}^{ATP}) in response to the changes in glucose uptake rate ($V_{glucose}$).

Independent variable:

- $V_{glucose}$ represent the glucose uptake rate (μmol mg protein^{-1} min^{-1})

Decision variables (model output):

- V_{glyc} and V_{resp} represent the rates of glycolysis or respiration (μmol mg protein^{-1} min^{-1}) utilized to maximize the ATP production rate in response to the glucose uptake rate.

The objective function is given by:

$$Maximize: \quad V_{total}^{ATP} = V_{glyc} \cdot \gamma_{glyc} + V_{resp} \cdot \gamma_{resp} \qquad [1]$$

The model must also satisfy three constraints that i) the rate of glycolysis and respiration cannot proceed faster than what is allowed by the proteome allocation for ATP-producing enzymes (ϕ_{total}^{ATP}) and ii) glucose uptake rate cannot be larger than the combined rates of glycolysis and respiration iii) the rate of the pathways cannot be negative (equation S1).

$$Subject\ to: \quad \frac{V_{glyc}}{V_{max}^{glyc}} + \frac{V_{resp}}{V_{max}^{resp}} \leq \phi_{total}^{ATP} \qquad [2]$$

$$V_{glyc} + V_{resp} \leq V_{glucose} \qquad [3]$$

$$V_{glyc} \geq 0, V_{resp} \geq 0 \qquad [S1]$$

We can use the V_{glyc} and V_{resp}values to calculate the absolute rates of glycolytic by-product (i.e. acetate, ethanol, or lactate) or oxygen consumption from the predicted rates of glycolysis and respiration (equation S2 and S3, respectively):

$$V_{glyc}^{byproduct} = \gamma_{gluc}^{byproduct} \cdot V_{glyc} \quad [S2]$$

$$V_{resp}^{O2} = \gamma_{resp}^{O2,consumed} \cdot V_{resp} \quad [S3]$$

This optimization problem can either be solved using linear programming or analytically using the Lagrange Dual Function (LDF) with Karush-Kuhn-Tucker (KKT) conditions. The benefit of this approach is to reveal how the constraints are governing the optimum. Therefore, we gain additional insight from the analytical solution for the biologically relevant cases. The KKT conditions provide necessary conditions for optimality problems with inequality constraints. The four conditions are known as (1) primal feasibility, (2) dual feasibility, (3) stationary, (4) complementary slackness.

(1) The primal feasibility condition simply means that for a solution to be considered valid, it must not violate any of the original given constraints. The requirement ensures the solution lies within the feasible region drawn by the constraints.

(2) The dual feasibility conditions states that the Lagrange multipliers associated with inequality constraints are non-negative. The requirement ensures that the optimized solution of the objective function will wither stay the same or decrease in value if the constraints are relaxed.

(3) The stationary condition states that at the optimal point, the gradient of the Lagrangian with respect to decision variables (model output) must be zero. In other words, it is not possible to increase or decrease the value of the objective function by adjusting these variables while still satisfying the constraints.

(4) The complimentary slackness condition states that the product of the Lagrangian multiplier and the associated inequality constraint must be zero. The complimentary slackness KKT condition allows us to determine when each constraint is in effect for a given set of inputs. If the inequality is not constraining the optimal solution, the associated Lagrangian multiplier will be zero so that it does not influence the optimality for the solution.

It is standard practice to use minimization rather than maximation in the presentation of LDF. Therefore, we convert the maximization into the equivalent minimization problem by multiplying the objective function by -1. We must also multiply constraint S1 by -1 so V_{glyc} and V_{resp} remain non-negative. With V_{max}^{glyc}, $\gamma_{glyc}, V_{max}^{resp}, \gamma_{resp} > 0,$ rewrite the program as:

$$\textit{Minimize}: \quad V_{total}^{ATP} = -(V_{glyc} \cdot \gamma_{glyc}) - (V_{resp} \cdot \gamma_{resp}) \quad [S4]$$

$$\textit{Subject to}: \quad \frac{V_{glyc}}{V_{max}^{glyc}} + \frac{V_{resp}}{V_{max}^{resp}} \leq \phi_{total}^{ATP} \quad [2]$$

$$V_{glyc} + V_{resp} \leq V_{glucose} \quad [3]$$

$$-V_{glyc} \leq 0, -V_{resp} \leq 0 \quad [S5]$$

Now, we form the Lagrangian function (L) by combing the objective function and constraints.
By combing the objective function and constraints, the Lagrangian function transforms a constrained optimization problem into an unconstrained one. The constraints here are weighted by the Lagrangian multipliers (λ), which gives us the change in value of objective function as the constraint changes, known as relaxing the constraint. Also, we assume $V_{max}^{glyc} \cdot \gamma_{glyc} \neq V_{max}^{resp} \cdot \gamma_{resp}$,

$V_{max}^{glyc} \neq V_{max}^{resp}$, $V_{max}^{resp} \cdot \gamma_{resp} \cdot V_{max}^{glyc} \neq V_{glyc} \cdot \gamma_{glyc} \cdot V_{max}^{resp}$, $\phi_{total}^{ATP} \cdot V_{max}^{glyc} \neq V_{glucose}$, $\phi_{total}^{ATP} \cdot V_{max}^{resp} \neq V_{glucose}$ to avoid redundancies. The Lagrangian function for this:

$$L = -(V_{glyc} \cdot \gamma_{glyc}) - (V_{resp} \cdot \gamma_{resp}) + \lambda_1 \left(\frac{V_{glyc}}{V_{max}^{glyc}} + \frac{V_{resp}}{V_{max}^{resp}} - \phi_{total}^{ATP}\right) + \lambda_2 \left(V_{glyc} + V_{resp} - V_{glucose}\right) - \lambda_{glyc} V_{glyc} - \lambda_{resp} V_{resp} \quad \text{[S6]}$$

Next, we take the partial derivative of the Lagrangian function with respect to V_{glyc} and V_{resp}. The Lagrange multipliers associated with the inequality constraints must be non-negative. Therefore, derivatives are given by:

$$\frac{\partial L}{\partial V_{glyc}} = -\gamma_{glyc} + \lambda_1 \frac{1}{V_{max}^{glyc}} + \lambda_2 - \lambda_{glyc} = 0 \quad \text{[S7]}$$

$$\frac{\partial L}{\partial V_{resp}} = -\gamma_{resp} + \lambda_1 \frac{1}{V_{max}^{resp}} + \lambda_2 - \lambda_{resp} = 0 \quad \text{[S8]}$$

For the inequality constraints, the complementary slackness for non-negative Lagrange is given by:

$$\lambda_1 > 0 \Rightarrow \frac{V_{glyc}}{V_{max}^{glyc}} + \frac{V_{resp}}{V_{max}^{resp}} = \phi_{total}^{ATP} \quad \text{[S9]}$$

$$\lambda_2 > 0 \Rightarrow V_{glyc} + V_{resp} = V_{glucose} \quad \text{[S10]}$$

$$\lambda_{glyc} > 0 \Rightarrow V_{glyc} = 0 \quad \text{[S11]}$$

$$\lambda_{resp} > 0 \Rightarrow V_{resp} = 0 \quad \text{[S12]}$$

We also know that in the optimal solution either $\frac{V_{glyc}}{V_{max}^{glyc}} + \frac{V_{resp}}{V_{max}^{resp}} = \phi_{total}^{ATP}$ or $V_{glyc} + V_{resp} = V_{glucose}$ or both (otherwise can improve by increasing V_{glyc} or V_{resp}) and we can use that. We can consider different cases, solve them, and see when they are optimal:

- $V_{glyc} > 0$, $V_{resp} = 0$, $\frac{V_{glyc}}{V_{max}^{glyc}} + \frac{V_{resp}}{V_{max}^{resp}} = \phi_{total}^{ATP}$ and $V_{glyc} + V_{resp} < V_{glucose}$

  In this case, $V_{glyc} = V_{max}^{glyc} \cdot \phi_{total}^{ATP}$, $\lambda_{glyc} = 0$, $\lambda_2 = 0$. Thus from (S7) $\lambda_1 = \gamma_{glyc} \cdot V_{max}^{glyc}$, and from (S8) $\lambda_{resp} = \gamma_{glyc} \cdot V_{max}^{glyc} - \gamma_{resp} \cdot V_{max}^{resp} > 0$.

  So for this solution to be legal we require: $V_{glucose} > \phi_{total}^{ATP} \cdot V_{max}^{glyc}$ (for second constraint) and $\gamma_{glyc} \cdot V_{max}^{glyc} - \gamma_{resp} \cdot V_{max}^{resp} > 0$ (for $\lambda_{resp} > 0$).

- $V_{glyc} > 0$, $V_{resp} = 0$, $\frac{V_{glyc}}{V_{max}^{glyc}} + \frac{V_{resp}}{V_{max}^{resp}} < \phi_{total}^{ATP}$ and $V_{glyc} + V_{resp} = V_{glucose}$.

  This is similar, $V_{glyc} = V_{glucose}$, $\lambda_{glyc} = 0$, $\lambda_1 = 0$. Thus from (S7) $\lambda_2 = \gamma_{glyc}$, and from (S8) $\lambda_{resp} = \gamma_{glyc} - \gamma_{resp}$. So for this solution to be legal we require:

  $V_{max}^{glyc} \cdot \phi_{total}^{ATP} > V_{glucose}$ (for first constraint) and $\gamma_{glyc} > \gamma_{resp}$ (for $\lambda_{resp} > 0$).

- $V_{glyc} = 0$, $\phi_{resp} > 0$, $\frac{V_{glyc}}{V_{max}^{glyc}} + \frac{V_{resp}}{V_{max}^{resp}} = \phi_{total}^{ATP}$ and $V_{glyc} + V_{resp} < V_{glucose}$.

  $\phi_{resp} = V_{max}^{resp} \cdot \phi_{total}^{ATP}$, and the same calculations as the first case, give:

  $V_{glucose} > \phi_{total}^{ATP} \cdot V_{max}^{resp}$ (for second constraint) and $\gamma_{resp} \cdot V_{max}^{resp} - \gamma_{glyc} \cdot V_{max}^{glyc} > 0$ (for

$\lambda_{glyc} > 0$).

- $V_{glyc} = 0$, $V_{resp} > 0$, $\frac{V_{glyc}}{V_{max}^{glyc}} + \frac{V_{resp}}{V_{max}^{resp}} < \phi_{total}^{ATP}$ and $V_{glyc} + V_{resp} = V_{glucose}$.
  $\phi_{resp} = V_{glucose}$ and the same calculations as second case, give:
  $V_{max}^{resp} \cdot \phi_{total}^{ATP} > V_{glucose}$(for first constraint) and $\gamma_{resp} > \gamma_{glyc}$ (for $\lambda_{glyc} > 0$).

- $\frac{V_{glyc}}{V_{max}^{glyc}} + \frac{V_{resp}}{V_{max}^{resp}} = \phi_{total}^{ATP}$ and $V_{glyc} + V_{resp} = V_{glucose}$. In this case, due to non-redundancy, we must have that both V_{glyc} and V_{resp} are non-zero. We can solve them:
  $$V_{glyc} = \frac{\phi_{total}^{ATP} \cdot V_{max}^{glyc} \cdot V_{max}^{resp} - V_{glucose} \cdot V_{max}^{glyc}}{V_{max}^{resp} - V_{max}^{glyc}},$$
  $$V_{resp} = \frac{V_{max}^{resp}(\phi_{total}^{ATP} \cdot V_{max}^{glyc} \cdot V_{max}^{resp} - V_{glucose})}{V_{max}^{glyc} - V_{max}^{resp}}$$

Note that neither can be zero by non-redundancy assumption, but they have to be positive, otherwise it's a violation of the non-negativity constraint. In this case, we have $\lambda_{glyc} = \lambda_{resp} = 0$ and λ_1, λ_2 can be positive. We can find them from (S9, S10):

$$\lambda_1 = \frac{V_{max}^{resp} \cdot V_{max}^{glyc}(\gamma_{resp} - \gamma_{glyc})}{V_{max}^{glyc} - V_{max}^{resp}}, \lambda_2 = \gamma_{glyc} - \frac{V_{max}^{resp}(\gamma_{resp} - \gamma_{glyc})}{V_{max}^{glyc} - V_{max}^{resp}}$$

Now to get the required positivity of ϕ_{resp}, ϕ_{glyc}, λ_1, λ_2, we have the following scenarios:

Either: $\phi_{total}^{ATP} \cdot V_{max}^{glyc} > V_{glucose} > \phi_{total}^{ATP} \cdot V_{max}^{resp}$ and $V_{max}^{glyc} \cdot \gamma_{glyc} > V_{max}^{resp} \cdot \gamma_{resp}$ and $V_{max}^{resp} \cdot \gamma_{resp} \cdot V_{max}^{glyc} > V_{max}^{glyc} \cdot \gamma_{glyc} \cdot V_{resp}$
OR: $\phi_{total}^{ATP} \cdot V_{max}^{glyc} < V_{glucose} < \phi_{total}^{ATP} \cdot V_{max}^{resp}$ and $\cdot V_{max}^{glyc} \cdot \gamma_{glyc} < V_{max}^{resp} \cdot \gamma_{resp}$ and $V_{max}^{resp} \cdot \gamma_{resp} \cdot V_{max}^{glyc} < \cdot V_{max}^{glyc} \cdot \gamma_{glyc} \cdot V_{max}^{resp}$

Finally, we would like to summarize the analytical solution below with the five cases. Our hope is that these equations as presented will be used in future efforts to study the resource allocation in energy metabolism.

If	$\gamma_{glyc} < \gamma_{resp}$<br>$V_{max}^{resp} \cdot \phi_{total}^{ATP} > V_{glucose}$	then	$V_{glyc} = 0, V_{resp} = V_{glucose}$	[4]
If	$\gamma_{glyc} < \gamma_{resp}$<br>$V_{max}^{glyc} \cdot \gamma_{glyc} > V_{max}^{resp} \cdot \gamma_{resp}$<br>$\phi_{total}^{ATP} \cdot V_{max}^{glyc} > V_{glucose} > \phi_{total}^{ATP} \cdot V_{max}^{resp}$	then	$V_{glyc} = \frac{\phi_{total}^{ATP} \cdot V_{max}^{glyc} \cdot V_{max}^{resp} - V_{glucose} \cdot V_{max}^{glyc}}{V_{max}^{resp} - V_{max}^{glyc}}$<br>$V_{resp} = \frac{V_{max}^{resp}(\phi_{total}^{ATP} \cdot V_{max}^{glyc} \cdot V_{max}^{resp} - V_{glucose})}{V_{max}^{glyc} - V_{max}^{resp}}$	[5]
If	$V_{max}^{glyc} \cdot \gamma_{glyc} > V_{max}^{resp} \cdot \gamma_{resp}$<br>$V_{max}^{glyc} \cdot \phi_{total}^{ATP} < V_{glucose}$	then	$V_{glyc} = V_{max}^{glyc} \cdot \phi_{total}^{ATP}, V_{resp} = 0$	[6]

If $\gamma_{glyc} > \gamma_{resp}$, $V_{max}^{glyc} \cdot \phi_{total}^{ATP} > V_{glucose}$ then $V_{glyc} = V_{glucose}, V_{resp} = 0$ [11]

If $\gamma_{glyc} > \gamma_{resp}$, $V_{max}^{glyc} \cdot \gamma_{glyc} < V_{max}^{resp} \cdot \gamma_{resp}$, $\phi_{total}^{ATP} \cdot V_{max}^{glyc} < V_{glucose} < \phi_{total}^{ATP} \cdot V_{max}^{resp}$ then

$$V_{glyc} = \frac{\phi_{total}^{ATP} \cdot V_{max}^{glyc} \cdot V_{max}^{resp} - V_{glucose} \cdot V_{max}^{glyc}}{V_{max}^{resp} - V_{max}^{glyc}}$$

$$V_{resp} = \frac{V_{max}^{resp}(\phi_{total}^{ATP} \cdot V_{max}^{glyc} \cdot V_{max}^{resp} - V_{glucose})}{V_{max}^{glyc} - V_{max}^{resp}} \quad [12]$$

If $V_{glyc} \cdot \gamma_{glyc} < V_{resp} \cdot \gamma_{resp}$, $V_{max}^{resp} \cdot \phi_{total}^{ATP} \leq V_{glucose}$ then $V_{glyc} = 0, V_{resp} = V_{max}^{resp} \cdot \phi_{total}^{ATP}$ [13]

Supplementary Discussion 2

Model with a Respiratory Substrate other than Glucose

We have extended our mathematical model to explore the effect of adding a respiratory substrate other than glucose. The only differences between the core model and extended model are that we decompose the original V_{resp} into a contribution from glucose V_{resp}^{gluc} and a contribution from alternative respiratory substrate V_{resp}^{sub}, and we add constraints to the core model to include these new terms to account for the maximal respiratory substrate uptake. We assumed that the specific activity of respiratory substrate utilization in units of µmol per mg cellular protein per min are the same for both glucose and the respiratory substrate as this value should be mostly set by the shared pathways of TCA cycle and ETC. Our simulations show that even in the presence of saturating respiratory substrate, there is no effect of additional respiratory substrate on the ratio of glycolysis and respiration used by the cell at all glucose uptake rates except for the very low glucose uptake rate when respiration is not saturated by glucose (*SI Appendix*, Fig. S2 *A* and *B*). Together, our results suggest that the excess of glucose that can be diverted to glycolysis, not the presence of a respiratory substrate, drive the Warburg Effect. We can use the same approach as Supplementary Discussion 1 to describe the additional uptake of a respiratory substrate. The linear program is given by:

$$Maximize: \quad V_{total}^{ATP} = \gamma_{glyc} \cdot V_{glyc} + \gamma_{resp} \cdot (V_{resp}^{gluc} + V_{resp}^{sub}) \qquad [S13]$$

$$Subject\ to: \quad \frac{V_{glyc}}{V_{max}^{glyc}} + \frac{1}{V_{max}^{resp}}(V_{resp}^{gluc} + V_{resp}^{sub}) \leq \phi_{total}^{ATP} \qquad [S14]$$

$$V_{glyc} + V_{resp}^{gluc} \leq V_{glucose} \qquad [S15]$$

$$V_{resp}^{sub} \leq V_{sub} \qquad [S16]$$

$$V_{glyc} \leq 0, V_{resp}^{gluc} \leq 0, V_{resp}^{sub} \leq 0 \qquad [S17]$$

where, $V_{resp}^{sub}$is the substrate respiration, and we assume both the yield (γ_{resp}) and the specific activity of the pathway, V_{max}^{resp}, are equal for respiration from glucose or an additional respiratory substrate. We also assume: $\gamma_{glyc} < \gamma_{resp}$ and $\gamma_{glyc} \cdot V_{max}^{glyc} > \gamma_{resp} \cdot V_{max}^{resp}$, which we have shown are necessary for eliciting the Warburg Effect (Fig. 1 *C-E*). These assumptions reduce the number of cases which need to be solved, decreasing the complexity of the analytical solution so that it in more interpretable. For all considered cases, we assume that $V_{sub} > 0.$ If $V_{sub} = 0$, the analytical solution yields identical cases as the original linear program as described in Supplementary Discussion 1.

The Lagrangian for this is given by:

$$L = -\left(\gamma_{glyc} \cdot V_{glyc}\right) - (\gamma_{resp} \cdot (V_{resp}^{gluc} + V_{resp}^{sub})) + \lambda_1(V_{glyc} + V_{resp}^{gluc} - V_{glucose}) + \lambda_2\left(\frac{V_{glyc}}{V_{max}^{glyc}} + \frac{V_{resp}^{gluc}}{V_{max}^{resp}} + \frac{V_{resp}^{sub}}{V_{max}^{resp}} - \phi_{total}^{ATP}\right) + \lambda_3(V_{resp}^{sub} - V_{sub}) - \lambda_{glyc}V_{glyc} - \lambda_{resp}^{gluc}V_{resp}^{gluc} - \lambda_{resp}^{sub}V_{resp}^{sub} \qquad [S18]$$

Lagrange multiplers are non-negative to account for the inequality constraints. Therefore, derivatives are given by:

$$\frac{\partial L}{\partial V_{glyc}} = -\gamma_{glyc} + \lambda_1 + \lambda_2\frac{1}{V_{max}^{glyc}} - \lambda_{glyc} = 0 \qquad [S19]$$

$$\frac{\partial L}{\partial V_{resp}^{gluc}} = -\gamma_{resp} + \lambda_1 + \lambda_2 \frac{1}{V_{max}^{resp}} - \lambda_{resp}^{gluc} = 0 \quad [S20]$$

$$\frac{\partial L}{\partial V_{resp}^{sub}} = -\gamma_{resp} + \lambda_2 \frac{1}{V_{max}^{resp}} + \lambda_3 - \lambda_{resp}^{sub} = 0 \quad [S21]$$

Complementary slackness for non-negative Lagrange multiplies:

$$\lambda_1 > 0 \Rightarrow V_{glyc} + V_{resp}^{gluc} = V_{glucose} \quad [S22]$$

$$\lambda_2 > 0 \Rightarrow \frac{V_{glyc}}{V_{max}^{glyc}} + \frac{V_{resp}^{gluc}}{V_{max}^{resp}} + \frac{V_{resp}^{sub}}{V_{max}^{resp}} = \phi_{total}^{ATP} \quad [S23]$$

$$\lambda_3 > 0 \Rightarrow V_{resp}^{sub} = V_{sub} \quad [S24]$$

$$\lambda_{glyc} > 0 \Rightarrow V_{glyc} = 0 \quad [S25]$$

$$\lambda_{resp}^{gluc} > 0 \Rightarrow V_{resp}^{gluc} = 0 \quad [S26]$$

$$\lambda_{resp}^{sub} > 0 \Rightarrow V_{resp}^{sub} = 0 \quad [S27]$$

The optimal solution needs to comply with all of these in addition to the original constraints. We know that in the optimal solution either $V_{glyc} + V_{resp}^{gluc} = V_{glucose}$ or $\frac{V_{glyc}}{V_{max}^{glyc}} + \frac{V_{resp}^{gluc}}{V_{max}^{resp}} + \frac{V_{resp}^{sub}}{V_{max}^{resp}} = \phi_{total}^{ATP}$ or both (otherwise can improve by increasing $V_{glyc}, V_{resp}^{gluc}$, or V_{resp}^{sub}). We consider the different cases in terms of glycolysis and respiration and see where they are optimal:

1. $V_{glyc} > 0, V_{resp}^{gluc} = 0$, or $V_{resp}^{sub} = 0$ (glycolysis only):
   First, we show that in this case we have that proteome allocation for ATP-producing enzymes is producing ATP at its maximum capacity. Thus, $V_{glyc} = \phi_{total}^{ATP} \cdot V_{max}^{glyc}$. And $\lambda_1 = \lambda_3 = \lambda_{glyc} = 0$, and consequently $\lambda_3 = \gamma_{glyc} \cdot V_{max}^{glyc}, \lambda_{resp}^{gluc} = \lambda_{resp}^{sub} = \gamma_{resp} - \gamma_{glyc} \cdot V_{max}^{glyc} - \gamma_{resp} \cdot V_{max}^{resp} > 0$.

2. $V_{glyc} > 0, V_{resp}^{gluc} > 0$, or $V_{resp}^{sub} = 0$ (glycolysis and respiration with glucose, no use of substrate):
   This case is not possible when $V_{sub} = 0$. It requires $\lambda_{glyc} = \lambda_{resp}^{gluc} = \lambda_3 = 0$ and then from (S21, S22) we get $\lambda_{resp}^{sub} = -\lambda_1$, which is only possible if $\lambda_{resp}^{sub} = \lambda_1 = 0$. In that case, we get: $\lambda_2 = \gamma_{glyc} \cdot V_{max}^{glyc} = \gamma_{resp} \cdot V_{max}^{resp}$ from $(S20, S21)$, which we know is not possible

3. $V_{glyc} > 0, V_{resp}^{gluc} = 0$, or $V_{resp}^{sub} > 0$ (glycolysis and respiration with substrate, no use of respiration with glucose):
   In this case, we get that glucose uptake rate constraint and proteome allocation for ATP-producing enzymes and utilized. The proteome allocation for ATP-producing enzymes is at its maximum capacity ($\frac{V_{glyc}}{V_{max}^{glyc}} + \frac{V_{resp}^{sub}}{V_{max}^{resp}} = \phi_{total}^{ATP}$). Otherwise, $\lambda_{glyc} = \lambda_2 = 0$, and therefore from (S19) $\lambda_1 = \gamma_{glyc}$ and from (S20) $\lambda_1 - \lambda_{resp}^{gluc} = \gamma_{resp}$, which contradicts $\gamma_{glyc} < \gamma_{resp}$. Glucose is fully used ($V_{glyc} = V_{glucose}$), because otherwise $\lambda_{glyc} = \lambda_1 = 0$ and from (S19) $\lambda_2 = \gamma_{glyc} \cdot V_{max}^{glyc}$ and from (S21) $\lambda_3 = \gamma_{resp} - \frac{\gamma_{glyc} \cdot V_{max}^{glyc}}{V_{max}^{resp}} < 0$, which is impossible.

If glucose is not abundant, $V_{glucose} < \phi_{total}^{ATP} \cdot V_{max}^{glyc}$, but we can fill the space with glycolysis and substrate respiration $\frac{V_{glyc}}{V_{max}^{glyc}} + \frac{V_{resp}^{sub}}{V_{max}^{resp}} \geq \phi_{total}^{ATP}$, then we have full glycolysis ($V_{glyc} = V_{glucose}$) and add substrate respiration $V_{resp}^{sub} = V_{max}^{resp} \cdot \left(\phi_{total}^{ATP} - \frac{V_{glucose}}{V_{max}^{glyc}}\right)$.

4. $V_{glyc} > 0, V_{resp}^{gluc} > 0$, or $V_{resp}^{sub} > 0$ (all active):
In this case too both Glucose and volume constraints must be full, and also the substrate must be fully used $V_{resp}^{sub} = V_{sub}$. This is because $\lambda_{glyc} = \lambda_{resp}^{gluc} = \lambda_{resp}^{sub} = 0$, the three equalities in (S19, S20, S21) can only hold simultaneously if $\lambda_1 > 0$, $\lambda_2 > 0$, $\lambda_3 > 0$. Note in this case we must also have that we cannot fill the proteome allocation for ATP-producing enzymes with just glycolysis and substrate, that is $\frac{1}{V_{max}^{glyc}} \cdot V_{glucose} + \frac{1}{V_{max}^{resp}} \cdot V_{sub} < \phi_{total}^{ATP}$, because $\frac{1}{V_{max}^{glyc}} \cdot V_{glyc} + \frac{1}{V_{max}^{resp}} \cdot V_{resp}^{gluc} > \frac{1}{V_{max}^{glyc}} \cdot V_{glucose}$ when $V_{glyc} + V_{resp}^{gluc} = V_{glucose}$.
Summary: If we cannot fill the space as before $\frac{1}{V_{max}^{glyc}} \cdot V_{glucose} + \frac{1}{V_{max}^{resp}} \cdot V_{sub} < \phi_{total}^{ATP}$, but we can fill it with respiration $\frac{1}{V_{max}^{resp}} (V_{glucose} + V_{sub}) > \phi_{total}^{ATP}$, then we have all three modes, use all glucose, and fill the volume, and $V_{resp}^{sub} = V_{sub}$ substrate is fully used.

5. $V_{glyc} = 0, V_{resp}^{gluc} > 0$, or $V_{resp}^{sub} > 0$ (no glycolysis):
In this case, it is easy to see glucose uptake rate and substrate uptake rate are fully used, that is: $V_{resp}^{gluc} = V_{glucose}$, $V_{resp}^{sub} = V_{sub}$. This is because $\lambda_{resp}^{gluc} = \lambda_{resp}^{sub} = 0$ and from (S20,S21) we see $\lambda_1 = \lambda_3$. If they are both 0, then λ_2 = b/e and then in (S19) we get: $\lambda_{glyc} = \frac{\gamma_{resp} \cdot V_{max}^{resp}}{V_{max}^{glyc}} - \gamma_{glyc} < 0$, and a contradiction. For the proteome allocation for ATP-producing enzymes constraint to hold this also requires that $\frac{1}{V_{max}^{resp}} (V_{glucose} + V_{sub}) \leq \phi_{total}^{ATP}$
Summary: If we cannot fill the proteome allocation for ATP-producing enzymes $\frac{1}{V_{max}^{resp}} (V_{glucose} + V_{sub}) \leq \phi_{total}^{ATP}$, then we maximize respiration using both sources $V_{resp}^{gluc} = V_{glucose}$, $V_{resp}^{sub} = V_{sub}$.

Supplemental Discussion 3

Model with Variable Oxygen Availability

All data used for parameter estimation and prediction validation in the following sections were collected under oxygen-rich conditions, therefore we assume oxygen saturation in our model. However, our model can also be extended to include an oxygen consumption constraint to simulate anaerobic or hypoxic environments (see *SI Appendix* for model formulation). In these oxygen limiting conditions, our model predicts that glycolysis will be utilized for ATP production at a lower glucose uptake rate as compared to oxygen-rich environments (*SI Appendix*, Fig S3). Furthermore, our model predicts the Pasteur Effect, where glycolysis is inhibited as oxygen becomes more available (*SI Appendix*, Fig S3)[30] and is well known to be driven by the maximization of the ATP production rate[31].

The linear program with oxygen constraint is given by:

$Maximize$: $$V_{ATP} = V_{glyc} \cdot \gamma_{glyc} + V_{resp} \cdot \gamma_{resp} \quad [1]$$

$Subject\ to$: $$\frac{V_{glyc}}{V_{max}^{glyc}} + \frac{V_{resp}}{V_{max}^{resp}} \leq \phi_{total}^{ATP} \quad [2]$$

$$V_{glyc} + V_{resp} \leq V_{glucose} \quad [3]$$

$$V_{resp} \cdot 6 \leq V_{O2} \quad [S28]$$

Supplementary Discussion 4

Derivation of Analytical Solution for *E.coli*, including the Pta-AckA Pathway

We can use the same approach to solve the more complex linear program for E. coli, which includes the Pta-AckA pathway as a third option The linear program is given by:

$$Maximize: \quad V_{ATP} = V_{ferm} \cdot \gamma_{ferm} + V_{pta} \cdot \gamma_{pta} + V_{resp} \cdot \gamma_{resp} \quad [24]$$

$$Subject\ to: \quad \frac{V_{ferm}}{V_{max}^{ferm}} + \frac{V_{pta}}{V_{max}^{pta}} + \frac{V_{resp}}{V_{max}^{resp}} \le \phi_{total}^{ATP} \quad [25]$$

$$V_{ferm} + V_{pta} + V_{resp} \le V_{glucose} \quad [26]$$

$$V_{ferm} \ge 0, V_{pta} \ge 0, V_{resp} \ge 0 \quad [S29]$$

We write the minimization program as:

$$Minimize: \quad V_{total}^{ATP} = -(V_{ferm} \cdot \gamma_{ferm}) - (V_{pta} \cdot \gamma_{pta}) - (V_{resp} \cdot \gamma_{resp}) \quad [S30]$$

$$Subject\ to: \quad \frac{V_{ferm}}{V_{max}^{ferm}} + \frac{V_{pta}}{V_{max}^{pta}} + \frac{V_{resp}}{V_{max}^{resp}} \le \phi_{total}^{ATP} \quad [S31]$$

$$V_{ferm} + V_{pta} + V_{resp} \le V_{glucose} \quad [S32]$$

$$-V_{ferm} \le 0, -V_{pta} \le 0, -V_{resp} \le 0 \quad [S33]$$

We assume $V_{max}^{ferm} \cdot \gamma_{ferm} \neq V_{max}^{pta} \cdot \gamma_{pta} \neq V_{max}^{resp} \cdot \gamma_{resp}$, $V_{max}^{ferm} \neq V_{max}^{pta} \neq V_{max}^{resp}$, $V_{max}^{resp} \cdot \gamma_{resp} \cdot V_{max}^{ferm} \neq V_{max}^{resp} \cdot \gamma_{resp} \cdot V_{max}^{pta} \neq V_{max}^{pta} \cdot \gamma_{pta} \cdot V_{max}^{ferm} \neq V_{max}^{pta} \cdot \gamma_{pta} \cdot V_{max}^{resp} \neq V_{ferm} \cdot \gamma_{ferm} \cdot V_{max}^{resp} \neq V_{max}^{ferm} \cdot \gamma_{ferm} \cdot V_{max}^{pta}$, $\phi_{total}^{ATP} \cdot V_{max}^{ferm} \neq V_{glucose}$, $\phi_{total}^{ATP} \cdot V_{max}^{pta} \neq V_{glucose,}$ $\phi_{total}^{ATP} \cdot V_{max}^{resp} \neq V_{glucose}$ to avoid redundancies. The Lagrangian function for this:

$$L = -(V_{ferm} \cdot \gamma_{ferm}) - (V_{pta} \cdot \gamma_{pta}) - (V_{resp} \cdot \gamma_{resp}) + \lambda_1 \left(\frac{V_{ferm}}{V_{max}^{ferm}} + \frac{V_{pta}}{V_{max}^{pta}} + \frac{V_{resp}}{V_{max}^{resp}} - \phi_{total}^{ATP}\right) + \lambda_2 \left(V_{ferm} + V_{resp} - V_{glucose}\right) - \lambda_{ferm} V_{ferm} - \lambda_{pta} V_{pta} - \lambda_{resp} V_{resp} \quad [S34]$$

We take the partial derivative of the Lagrangian function with respect to V_{ferm}, $V_{pta,}$ and V_{resp}. The Lagrange multipliers associated with the inequality constraints must be non-negative. Therefore, derivatives are given by:

$$\frac{\partial L}{\partial V_{ferm}} = -\gamma_{ferm} + \lambda_1 \frac{1}{V_{max}^{ferm}} + \lambda_2 - \lambda_{ferm} = 0 \quad [S35]$$

$$\frac{\partial L}{\partial V_{pta}} = -\gamma_{pta} + \lambda_1 \frac{1}{V_{max}^{pta}} + \lambda_2 - \lambda_{pta} = 0 \quad [S36]$$

$$\frac{\partial L}{\partial V_{resp}} = -\gamma_{resp} + \lambda_1 \frac{1}{V_{max}^{resp}} + \lambda_2 - \lambda_{resp} = 0 \quad [S37]$$

For the inequality constraints, the complementary slackness for non-negative Lagrange is given by:

$$\lambda_1 > 0 \Rightarrow \frac{V_{glyc}}{V_{max}^{glyc}} + \frac{V_{resp}}{V_{max}^{resp}} = \phi_{total}^{ATP} \quad [S38]$$

$$\lambda_2 > 0 \Rightarrow V_{glyc} + V_{resp} = V_{glucose} \quad [S39]$$

$$\lambda_{ferm} > 0 \Rightarrow V_{ferm} = 0 \quad [S40]$$

$$\lambda_{pta} > 0 \Rightarrow V_{pta} = 0 \quad [S41]$$

$$\lambda_{resp} > 0 \Rightarrow V_{resp} = 0 \quad [S42]$$

We also know that in the optimal solution either $\frac{V_{ferm}}{V_{max}^{ferm}} + \frac{V_{pta}}{V_{max}^{pta}} + \frac{V_{resp}}{V_{max}^{resp}} = \phi_{total}^{ATP}$ or $V_{ferm} + V_{pta} + V_{resp} = V_{glucose}$ or both (otherwise can improve by increasing V_{glyc} or V_{resp}) and we can use that. We can consider different cases, solve them, and see when they are optimal. Note we only present the three relevant cases to demonstrate that given the relationship between the yield (i.e. $\gamma_{ferm} < \gamma_{pta} < \gamma_{resp}$) and the specific activity (i.e. $\gamma_{ferm} < \gamma_{resp} < \gamma_{pta}$) of the three pathways:

- $V_{ferm} = 0$, $V_{pta} > 0$, $V_{resp} = 0$, $\frac{V_{ferm}}{V_{max}^{ferm}} + \frac{V_{pta}}{V_{max}^{pta}} + \frac{V_{resp}}{V_{max}^{resp}} = \phi_{total}^{ATP}$ and $V_{ferm} + V_{pta} + V_{resp} < V_{glucose}$
  In this case, $V_{pta} = V_{max}^{pta} \cdot \phi_{total}^{ATP}$, $\lambda_{pta} = 0$, $\lambda_2 = 0$. Thus from (S35) $\lambda_1 = \gamma_{pta} \cdot V_{max}^{pta}$, and from (S36) $\lambda_{resp} = \gamma_{ferm} \cdot V_{max}^{ferm} - \gamma_{resp} \cdot V_{max}^{resp} > 0$ and (S37) $\lambda_{pta} = \gamma_{ferm} \cdot V_{max}^{ferm} - \gamma_{pta} \cdot V_{max}^{pta} > 0$
  So for this solution to be legal we require: $V_{glucose} > \phi_{total}^{ATP} \cdot V_{max}^{ferm}$ (for second constraint), $\gamma_{ferm} \cdot V_{max}^{ferm} - \gamma_{resp} \cdot V_{max}^{resp} > 0$ (for $\lambda_{resp} > 0$), and $\gamma_{ferm} \cdot V_{max}^{ferm} - \gamma_{pta} \cdot V_{max}^{pta} > 0$ (for $\lambda_{pta} > 0$).

- $V_{ferm} = 0$, $V_{pta} = 0$, $V_{resp} > 0$, $\frac{V_{ferm}}{V_{max}^{ferm}} + \frac{V_{pta}}{V_{max}^{pta}} + \frac{V_{resp}}{V_{max}^{resp}} < \phi_{total}^{ATP}$ and $V_{ferm} + V_{pta} + V_{resp} = V_{glucose}$
  In this case, $\phi_{resp} = V_{glucose}$, $\lambda_{resp} = 0$, $\lambda_1 = 0$. Thus from (S35) $\lambda_2 = \gamma_{resp}$, and from (S36) $\lambda_{ferm} = \gamma_{resp} - \gamma_{ferm}$ and from (S37) $\lambda_{pta} = \gamma_{resp} - \gamma_{pta}$. So for this solution to be legal we require:
  $V_{max}^{resp} \cdot \phi_{total}^{resp} > V_{glucose}$ (for first constraint) and $\gamma_{resp} > \gamma_{ferm}$ (for $\lambda_{ferm} > 0$) and $\gamma_{resp} > \gamma_{pta}$ (for $\lambda_{pta} > 0$).

- $V_{ferm} = 0$, $V_{pta} > 0$, $V_{resp} > 0$, $\frac{V_{glyc}}{V_{max}^{glyc}} + \frac{V_{resp}}{V_{max}^{resp}} = \phi_{total}^{ATP}$ and $V_{glyc} + V_{resp} = V_{glucose}$.
  $V_{pta} = \frac{\phi_{total}^{ATP} \cdot V_{max}^{pta} \cdot V_{max}^{resp} - V_{glucose} \cdot V_{max}^{pta}}{V_{max}^{resp} - V_{max}^{pta}}, V_{resp} = \frac{V_{max}^{resp}(\phi_{total}^{ATP} \cdot V_{max}^{glyc} \cdot V_{max}^{resp} - V_{glucose})}{V_{max}^{glyc} - V_{max}^{resp}}$

Finally, we would like to summarize the analytical solution below with the above three cases:

$$\text{If} \quad \begin{matrix} \gamma_{ferm} < \gamma_{resp} \\ \gamma_{pta} < \gamma_{resp} \\ V_{max}^{resp} \cdot \phi_{total}^{ATP} > V_{glucose} \end{matrix} \quad \text{then} \quad V_{ferm} = 0, V_{pta} = 0, V_{resp} = V_{glucose} \quad [27]$$

If

$$\gamma_{ferm} < \gamma_{resp}$$

$$\gamma_{pta} < \gamma_{resp}$$

$$V_{max}^{ferm} \cdot \gamma_{ferm} < V_{max}^{pta} \cdot \gamma_{pta}$$

$$V_{max}^{pta} \cdot \gamma_{pta} > V_{max}^{resp} \cdot \gamma_{resp}$$

$$\phi_{total}^{ATP} \cdot V_{max}^{glyc} > V_{glucose} > \phi_{total}^{ATP} \cdot V_{max}^{resp}$$

then

$$V_{ferm} = 0$$

$$V_{pta} = \frac{\phi_{total}^{ATP} \cdot V_{max}^{pta} \cdot V_{max}^{resp} - V_{glucose} \cdot V_{max}^{pta}}{V_{max}^{resp} - V_{max}^{pta}}$$

$$V_{resp} = \frac{V_{max}^{resp}(\phi_{total}^{ATP} \cdot V_{max}^{pta} \cdot V_{max}^{resp} - V_{glucose})}{V_{max}^{pta} - V_{max}^{resp}} \quad [28]$$

If

$$V_{max}^{ferm} \cdot \gamma_{ferm} < V_{max}^{pta} \cdot \gamma_{pta}$$

$$V_{max}^{pta} \cdot \gamma_{pta} > V_{max}^{resp} \cdot \gamma_{resp}$$

$$V_{max}^{pta} \cdot \phi_{total}^{ATP} < V_{glucose}$$

then

$$V_{ferm} = 0, V_{pta} = V_{max}^{glyc} \cdot \phi_{total}^{ATP}, V_{resp} = 0 \quad [29]$$

Supplementary Discussion 5

Model Predictions of ATP production with cycloheximide treatment

Elsemann et al. 2022 treated *S. cerevisae* growing in glucose-excess batch culture with cycloheximide, a translation inhibitor. Our analysis of the proteomics data reveals changes in the ATP-producing proteome space (ϕ_{total}^{ATP}) with cycloheximide treatment (*SI Appendix*, Fig. S9*A*). More importantly, we found a strong linear correlation between the glucose uptake rate and ϕ_{total}^{ATP} (ρ = 0.94) during cycloheximide treatment (*SI Appendix*, Fig. S9*B*). We use the slope and intercept of this linear relationship to inform our model about how the ϕ_{total}^{ATP} expands with the glucose uptake rate. If this expansion in not accounted for (i.e. ϕ_{total}^{ATP} is held constant), our model predicts decrease in respiration as the glucose uptake rate increases (*SI Appendix*, Fig. S9*C*). However, by incorporating the empirically derived ϕ_{total}^{ATP} expansion rate, our model can predict glycolysis and respiration rates across the cycloheximide treatments (*SI Appendix*, Fig. S9*D*). Here, we observe a sustained utilization of respiration as an ATP-producing strategy due to the changing ATP-producing space (ϕ_{total}^{ATP}) as a result of cycloheximide treatment.

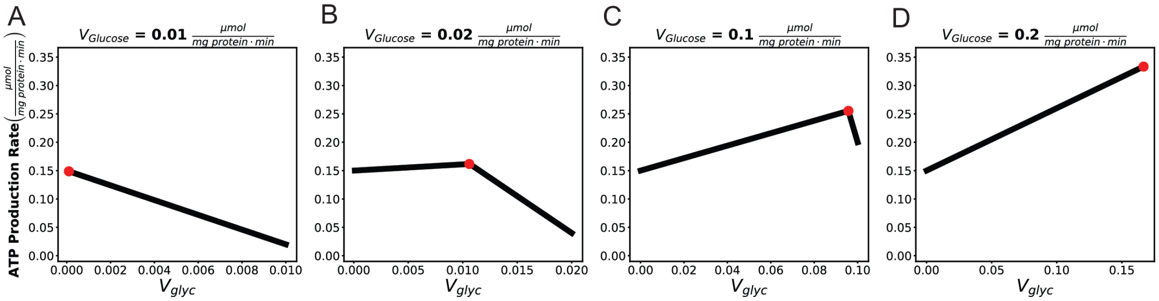

Fig S1. Model predictions under varying glucose uptake rates. (*A*) Rate of glycolysis that yields the highest ATP production rate (highlighted with a red dot) for a glucose uptake rate $V_{glucose}$ = 0.01 µmol per mg cellular protein per min. (*B*) $V_{glucose}$ = 0.02 µmol per mg cellular protein per min. (*C*) $V_{glucose}$ = 0.1 µmol per mg cellular protein per min. (*D*) $V_{glucose}$ = 0.2 µmol per mg cellular protein per min.

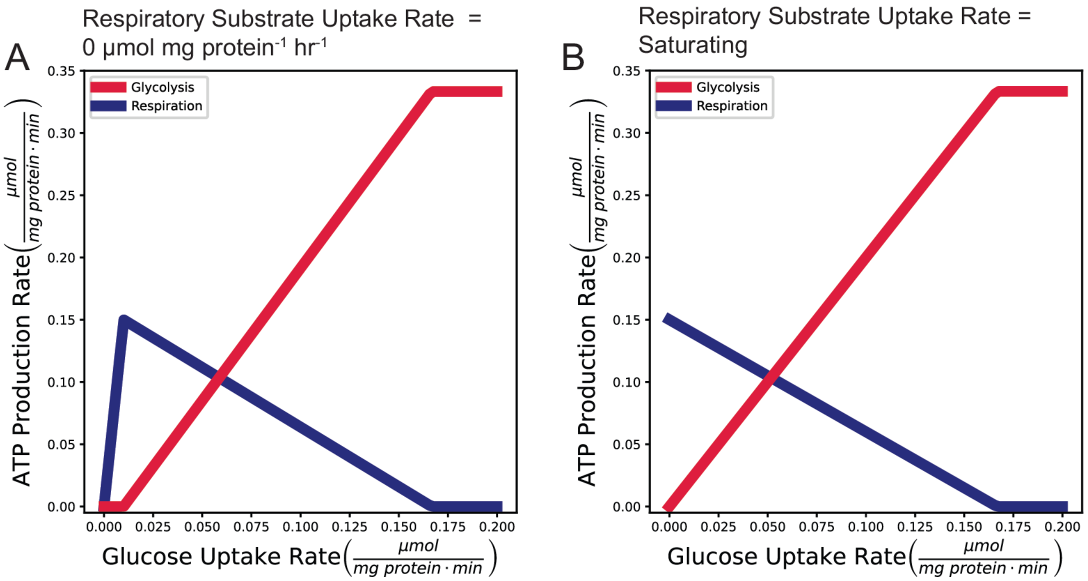

Fig. S2. Model predictions under varying glucose and respiratory substrate uptake rates. (*A*) ATP production rates of glycolysis (red) and respiration (blue) in the absence of respiratory substrate and (*B*) in the presence of saturating respiratory substrate.

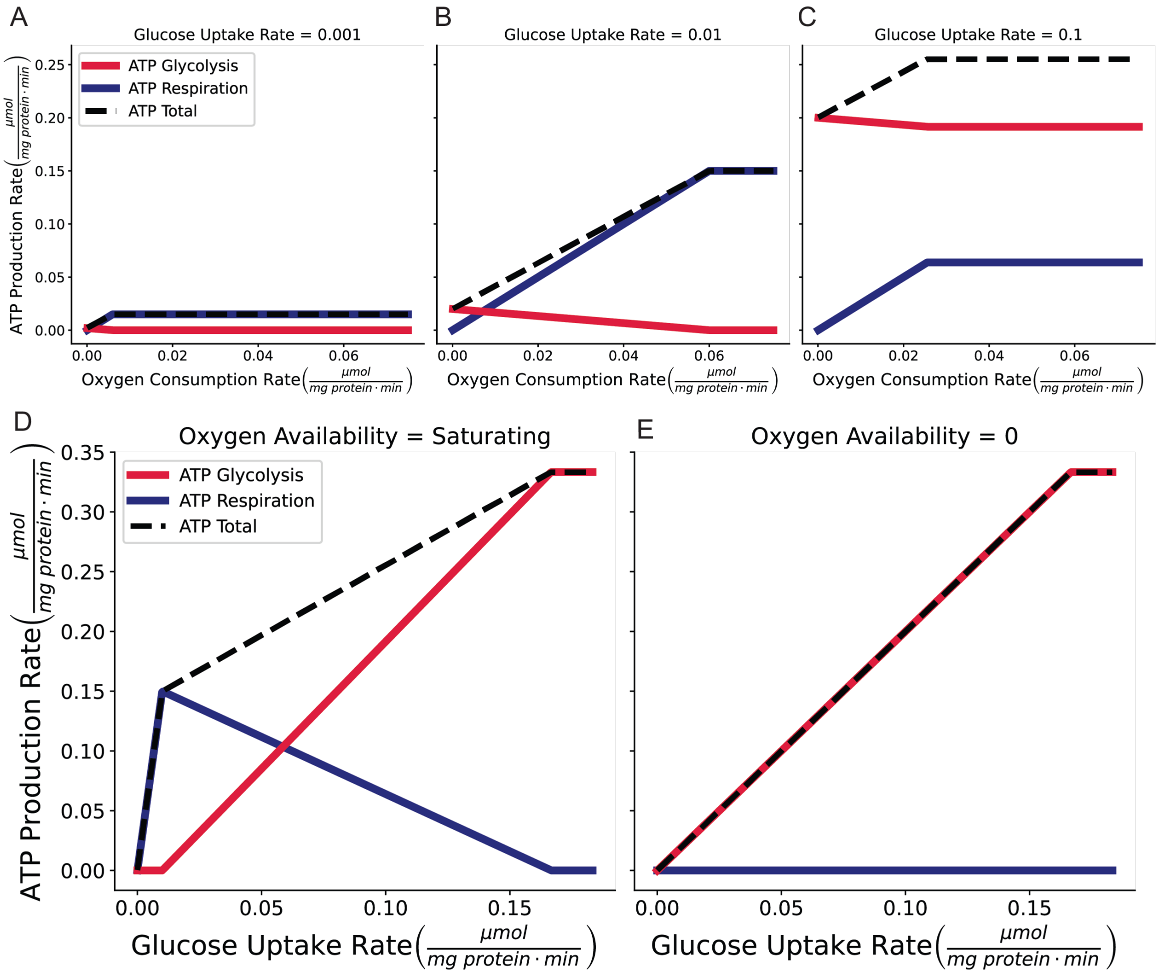

Fig. S3. Model predictions under varying oxygen availabilities. (*A*) ATP production rates of glycolysis (red) and respiration (blue) as the oxygen consumption rate increases for glucose uptake rate of 0.001 (*B*) 0.01 (*C*) 0.1 (μmol mg protein^{-1} min^{-1}) and (*D*) ATP production rates of glycolysis (red) and respiration (blue) in response to increased glucose uptake rates in the presence of saturating oxygen availability (*E*) in the absence of oxygen.

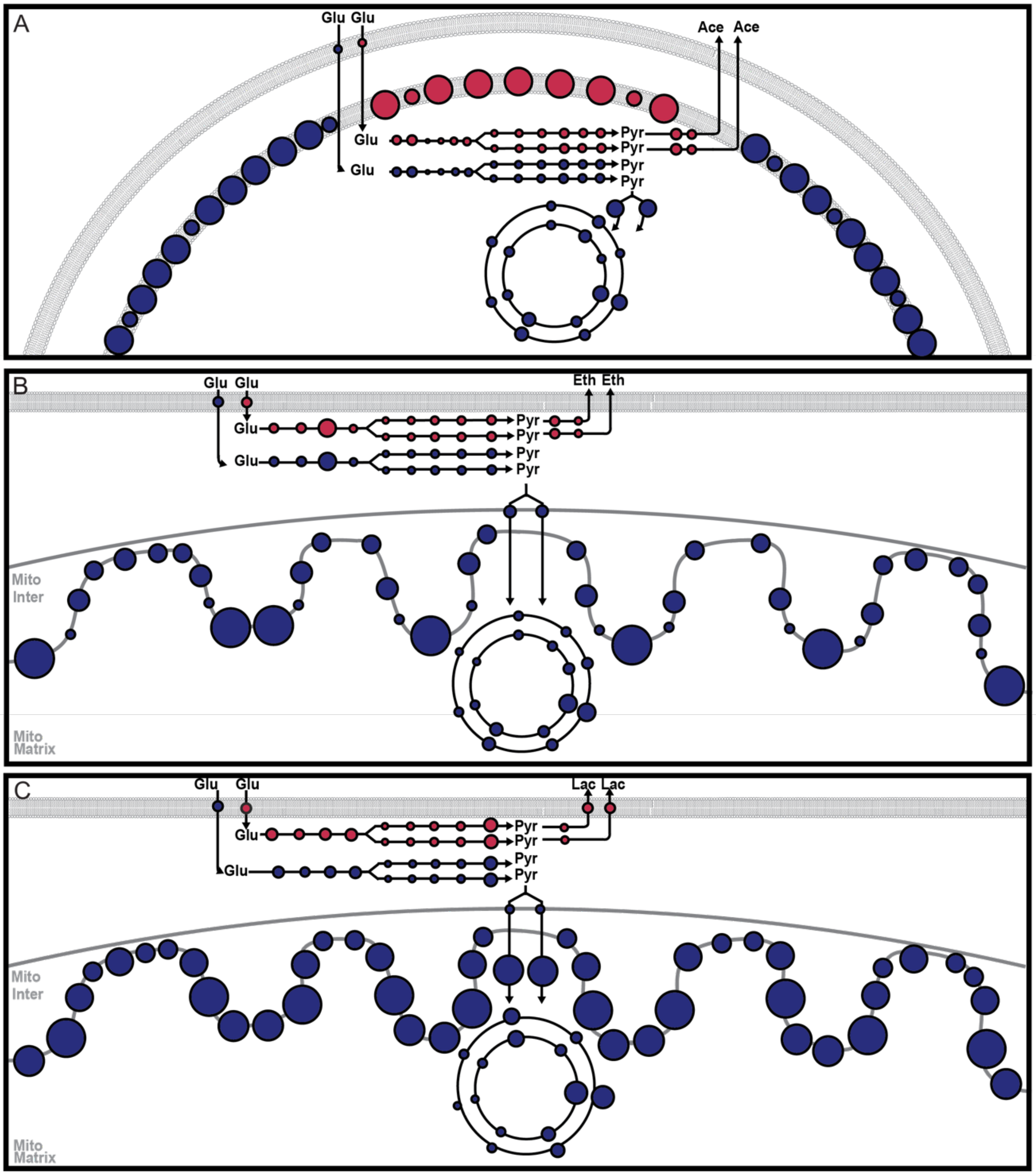

Fig. S4. Glycolysis and respiration pathway sizes in *E. coli*, *S. cerevisiae* and mammals. (*A*-*C*), Glycolysis (red) and respiration (blue) pathway outline for (*A*) *E. coli* (respiro-fermentative Pta-AckA pathway that uses both glycolysis and ETC is displayed), (*B*) *S. cerevisiae*, and (*C*) mammalian cells. Size of circle is proportional to the MW of each enzyme in the pathway. Furthermore, the pathway stoichiometry is represented under the assumption that each enzyme catalyzes its reaction at the same rate.

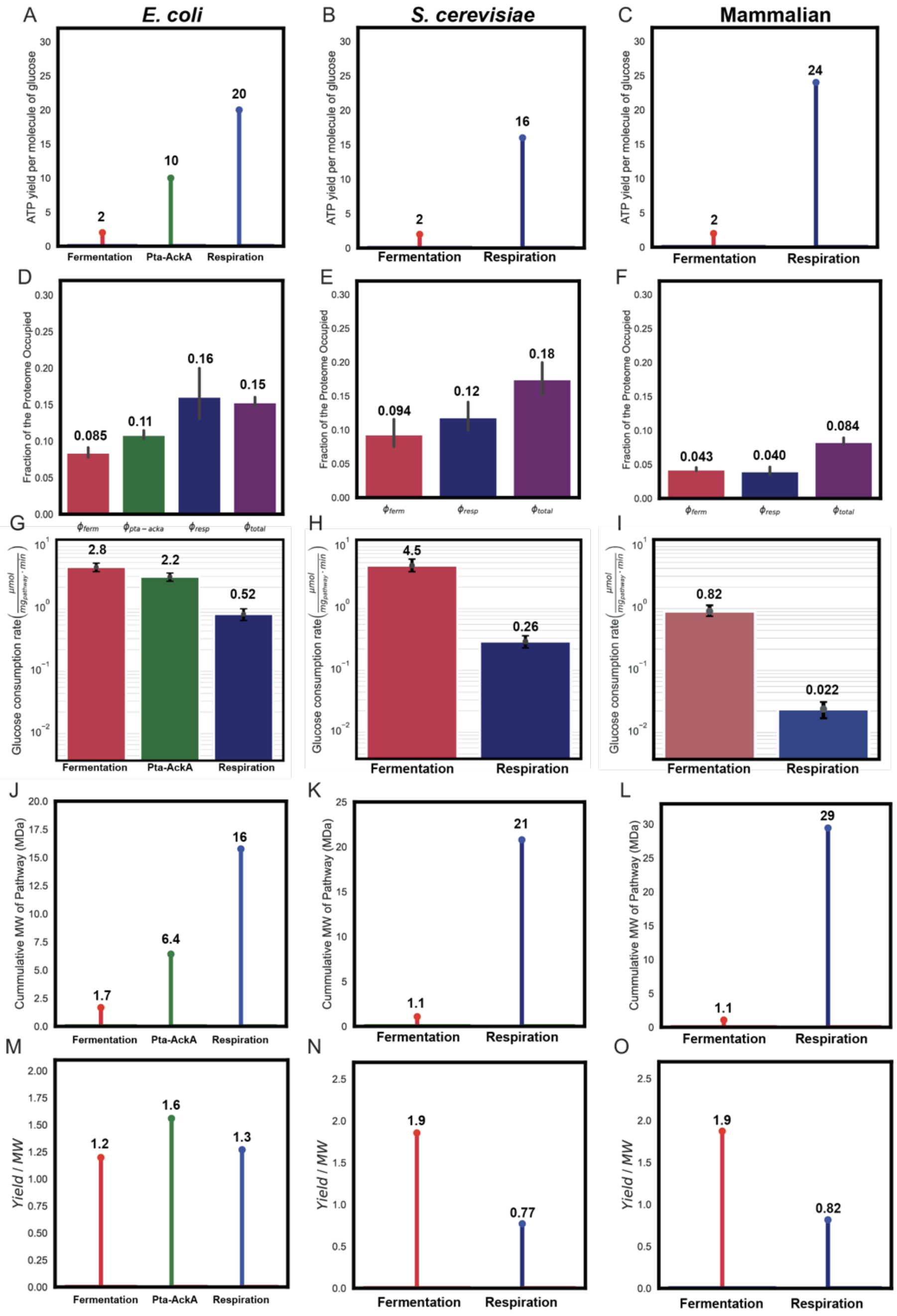

Fig. S5. Parameter estimates for *E. coli*, *S. cerevisiae*, and mammalian cells. (*A*-*C*) ATP yield per molecule of glucose for fermentation (red) and respiration (blue) for *E. coli*, *S. cerevisiae*, and mammalian cells, respectively. (*D*-*F*), The fraction of the proteome occupied by enzymes for fermentative glycolysis (red), Pta-AckA glycolysis (green), respiration (blue), and both pathways (purple) for *E. coli*, *S. cerevisiae*, and mammalian cells, respectively. For *E. coli* and *S. cerevisiae*, the glycolysis and respiration estimates represent proteome occupancies for cells grown in batch culture with non-limiting glucose or non-fermentative substrate, respectively. Error bars are 95 percent confidence interval. (*G*-*I*) Maximal observed glucose uptake rate (μmol per mg cellular protein per min) for fermentative glycolysis (red) and respiration (blue) for *E. coli*, *S. cerevisiae*, and mammalian cells, respectively. Error bars are 95 percent confidence interval calculated from 10000 bootstrap iterations. (*J*-*L*) Cumulative molecular weight (MDa) of each Pathway (*M*-*O*)**,** Ratio of the ATP yield per molecule of glucose to molecular weight (MDa) for each pathway.

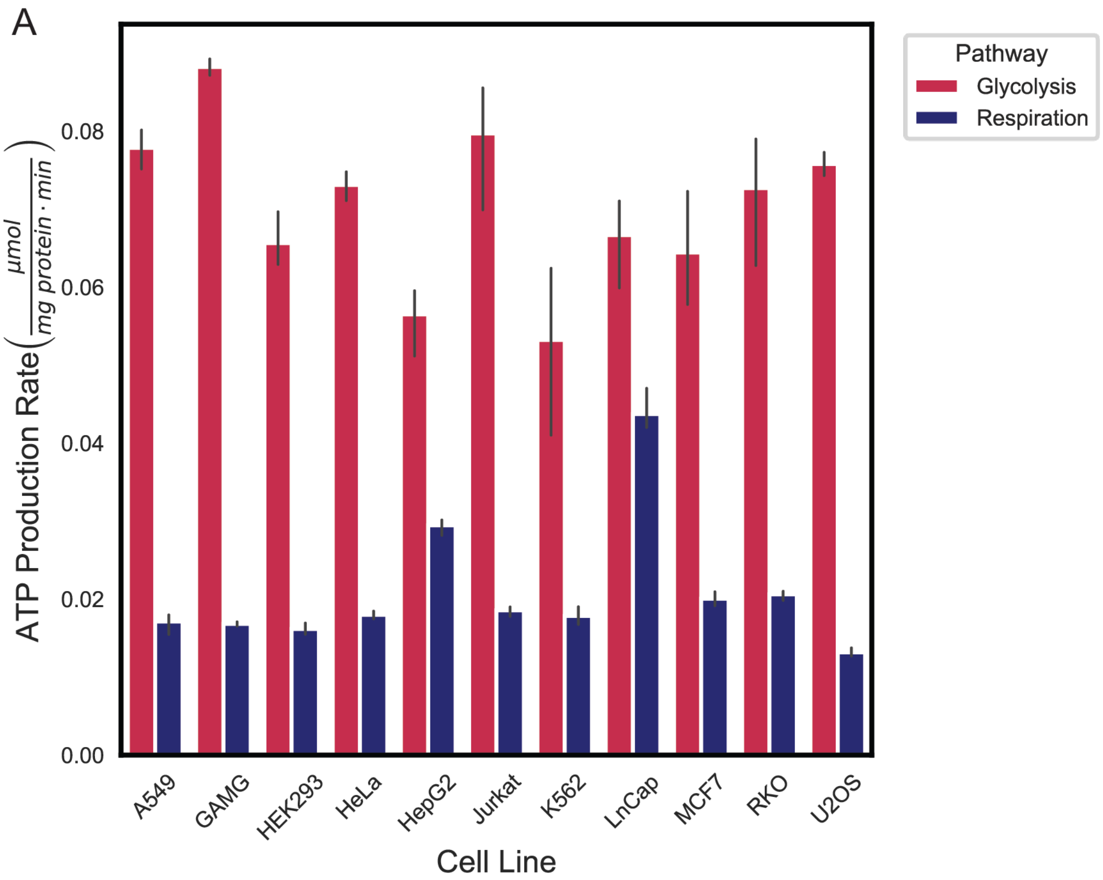

Fig. S6. Cell line-specific maximal ATP production rate capacities of glycolysis and respiration in mammalian cells. (*A*) Estimates of maximal ATP production rate (μmol mg protein^{-1} min^{-1}) for fermentative glycolysis (red) and respiration (blue) for 11 mammalian cells lines using proteomics data from Geiger et al. 2012.

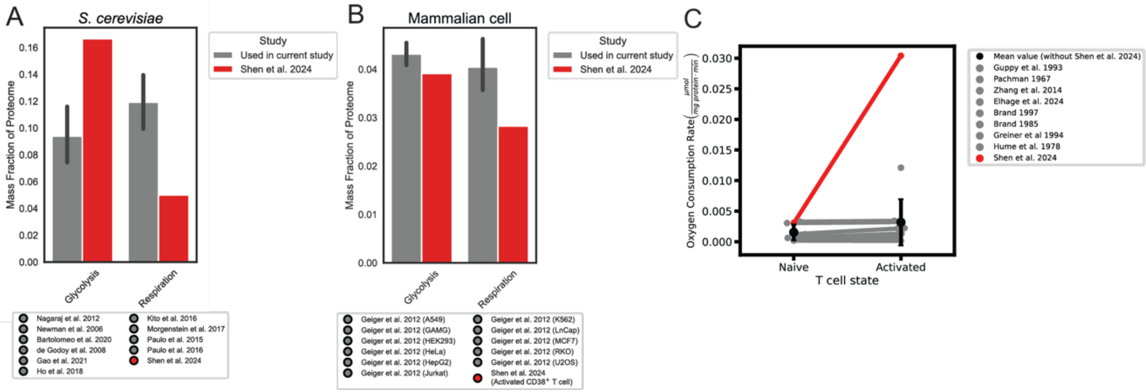

Fig. S7. Parameter differences in the current study and Shen et al. 2024. (*A*) Proteome occupancy for glycolysis and respiration in *S. cerevisiae* on ten previous reports that we used in our study (grey) and Shen et al. 2024 (red). The two-fold difference in both glycolysis and respiration occupancy is sufficient to account for the reported differences of proteome efficiency between our studies. (*B*) Proteome occupancy for glycolysis and respiration in mammalian cells in our study (grey) compared to Shen et al. 2024 (red). The differences in proteome occupancy are not sufficient to account for the reported differences of proteome efficiency. (*C*) Oxygen consumption in naïve and activated T-cells reported in Shen et al. 2024 (red) as compared to eight other studies (grey), which is likely the principal difference leading to our divergent conclusions for mammalian cells.

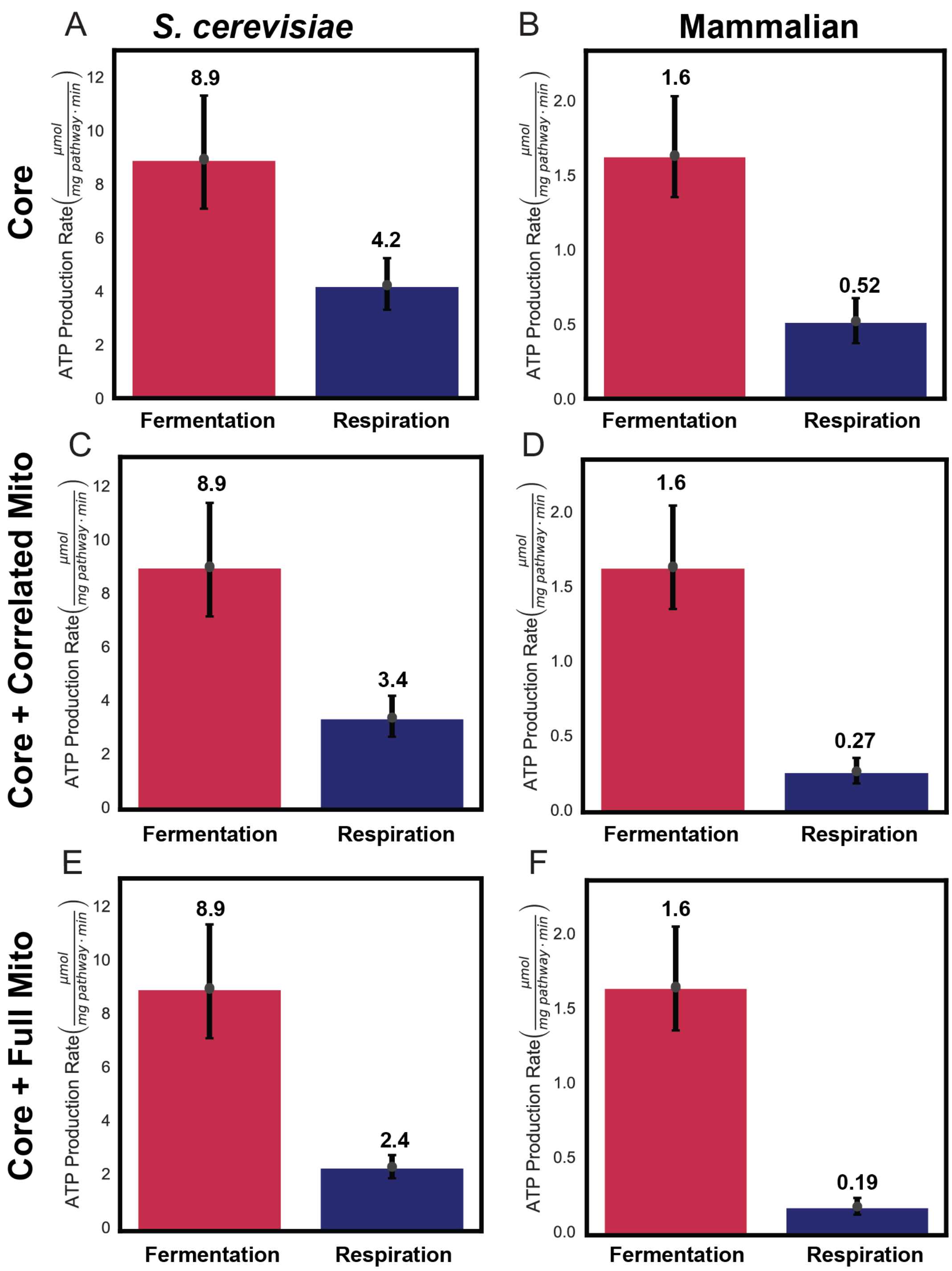

Fig. S8. Pathway-specific ATP production rates for *S. cerevisiae* and mammalian cells with additional mitochondrial proteins. (*A* and *F*) Maximal ATP production rate (μmol mg pathway^{-1} min^{-1}) for fermentative glycolysis (red) and respiration (blue) without any additional mitochondrial proteins (Fig. 2 *B* and *C* in the main text) (*A* and *B*) with inclusion of positively and significantly correlated mitochondrial proteins from (*C* and *D*) and all mitochondrial proteins (*E* and *F*) for *S. cerevisiae* (*A* and *C* and *E*) and mammalian cells (*B* and *D* and *F*). Error bars are the 95 percent confidence interval calculated from 10,000 bootstrap iterations.

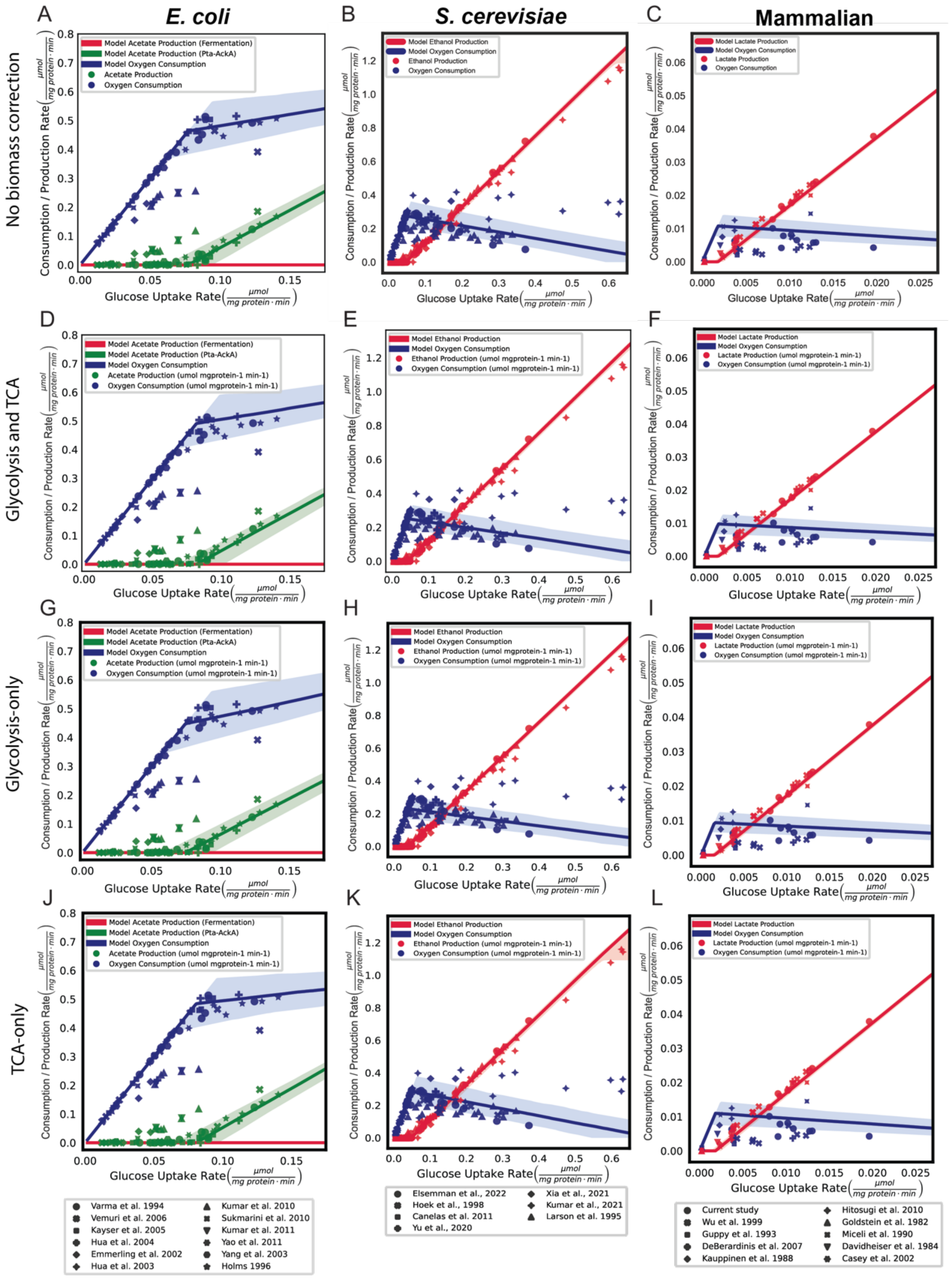

Fig. S9. Organism-specific model predictions of ATP production using different contributions towards biomass production. (*A-C*) Model prediction of fermentative glycolysis and respiration rates with no correction for contribution of glycolysis or TCA enzymes (Fig. 3 *A-C* in the main text), (*D-F*) a 30% correction for both glycolysis and the TCA cycle enzymes, (*G-I*) a 30% correction for only glycolysis enzymes, or (*J-L*) a 30% correction for only TCA cycle enzymes towards biomass production.

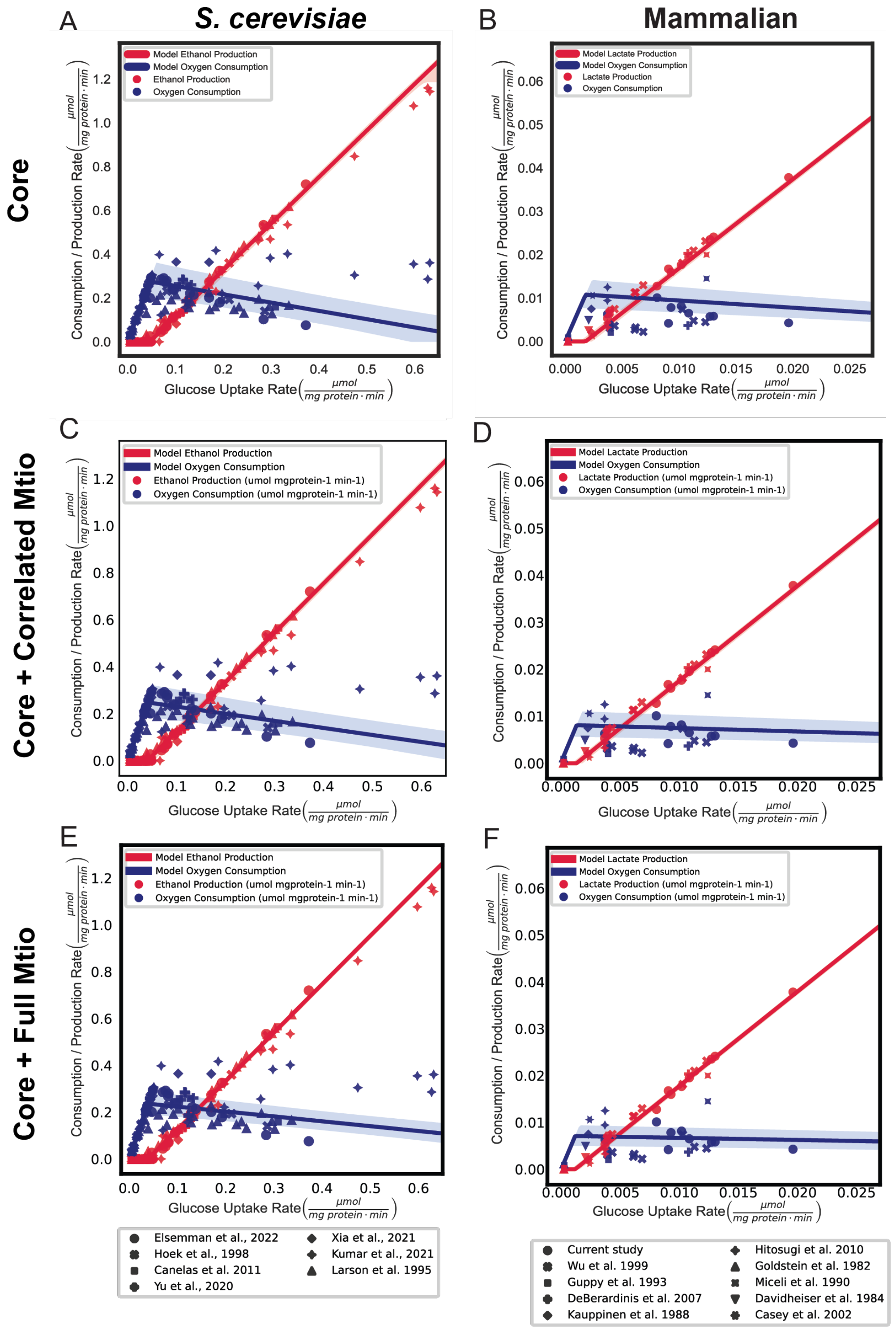

Fig. S10. Organism-specific model predictions of ATP production using different inclusion criteria for mitochondrial proteins. (A-F) Model prediction of fermentative glycolysis and respiration rates with only mitochondrial proteins that are directly involved in respiration (Fig. 3 *B* and *C* in the main text) (*A* and *B*) with inclusion of additional proteins that are positively and significantly correlated with mitochondrial proteins from (*C* and *D*) and all mitochondrial proteins (*E* and *F*) for *S. cerevisiae* (*A* and *C* and *E*) and mammalian cells (*B* and *D* and *F*).

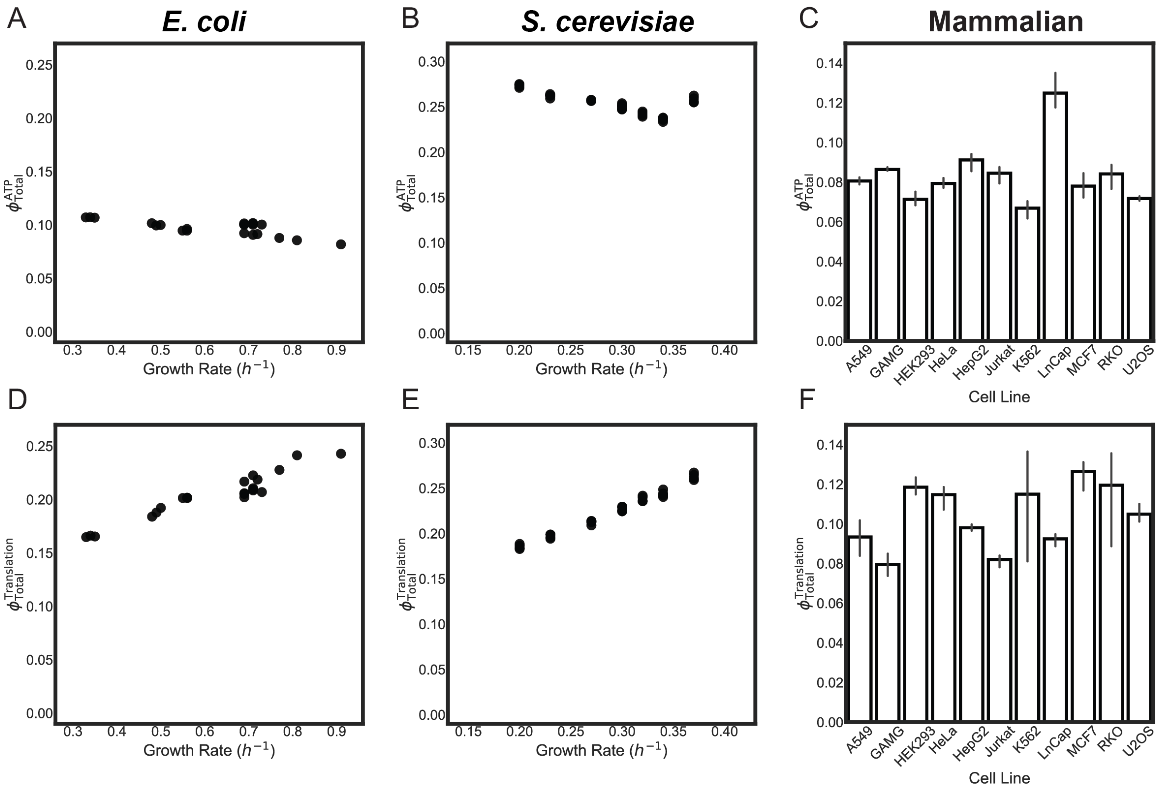

Fig. S11. Conservation of ATP-producing proteome allocation and variation in translational proteome allocation. (*A* and *B*) Fraction of ATP-producing enzymes across with increasing growth rates in *E. coli* and *S. cerevisiae*., respectively. (*C*) Fraction of ATP-producing enzymes for various mammalian cell lines. c, Fraction of ATP-producing enzymes across cell lines. (*D* and *E*) Fraction of translational enzymes across with increasing growth rates in *E. coli* and *S. cerevisiae*., respectively. (*F*) Fraction of translational enzymes for various mammalian cell lines. *E. coli*, *S. cerevisiae*, and mammalian cell data is from Mori et al. 2022, Elsemann et al. 2022, and Geiger et al. 2012, respectively.

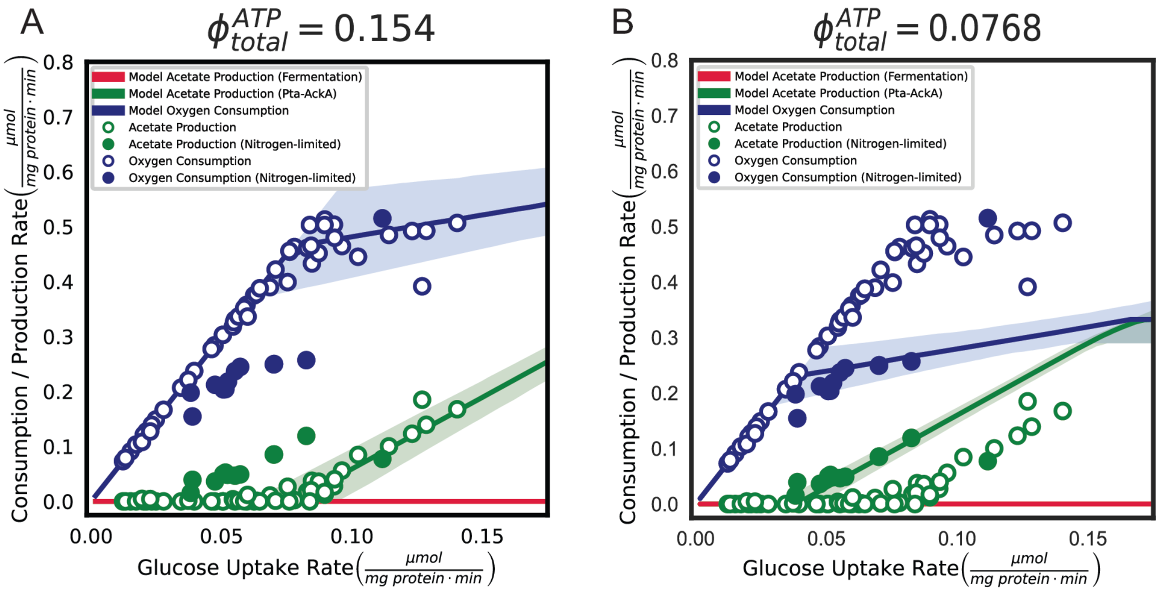

Fig. S12. Model predictions of ATP production under nitrogen limitation *(A)* Model predictions of acetate production and oxygen consumption with is ϕ^{ATP}_{total} = 0.154, which represents the mean value derived from our experimental proteomics data. *(B)* Model predictions of acetate production and oxygen consumption with is ϕ^{ATP}_{total} = 0.0768, reflecting a two-fold decrease in the allocation of proteome space to ATP-producing enzymes.

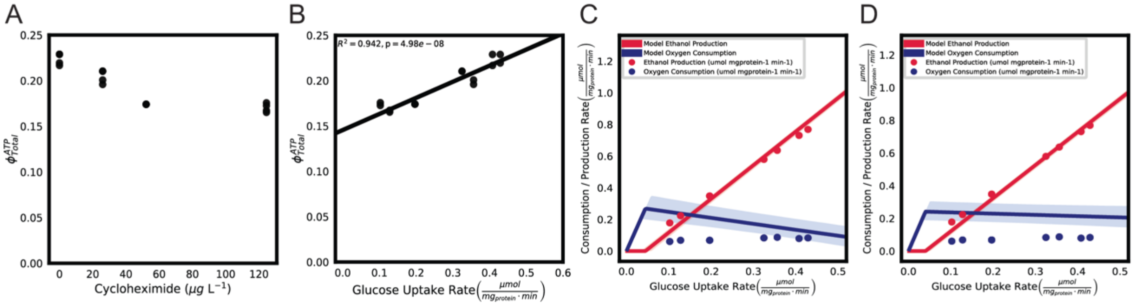

Fig. S13. Model predictions of ATP production with cycloheximide treatment. (*A*) Changes in ϕ_{total}^{ATP} with cycloheximide treatment in *S. cerevisiae*. (*B*) Linear regression of the glucose uptake rate (μmol per mg cellular protein per min) on ϕ_{total}^{ATP} with cycloheximide treatment. (*C*) Model prediction of glycolysis and respiration rates when ϕ_{total}^{ATP} is held constant with the glucose uptake rate. (*D*) Model prediction of glycolysis and respiration rates when ϕ_{total}^{ATP} increases with the glucose uptake rate. Data is from Elsemann et al. 2022.
